# Synthesis and characterization of electroactive chitosan/gelatin/PEDOT:PSS hydrogels with mixed ionic–electronic conductivity for potential wound healing applications

**DOI:** 10.1039/d5ra09790h

**Published:** 2026-02-09

**Authors:** Dania Adila Ahmad Ruzaidi, Mashani Mohamad, Norita Salim, Zarif Mohamed Sofian, Nur Hidayah Shahemi, Hazwanee Osman, Rosmamuhamadani Ramli, Mohamed Izzharif Abdul Halim, Mohd Ifwat Mohd Ghazali, Kishor Kumar Sadasivuni, Mohd Muzamir Mahat

**Affiliations:** a Faculty of Applied Sciences, Universiti Teknologi MARA Shah Alam Campus 40450 Shah Alam Malaysia mmuzamir@uitm.edu.my; b Faculty of Applied Sciences, Universiti Teknologi MARA Cawangan Perak, Tapah Campus 35400 Tapah Road Malaysia; c Department of Pharmaceutical Life Sciences, Faculty of Pharmacy, Universiti Teknologi MARA (UiTM) 42300 Bandar Puncak Alam Selangor Malaysia; d Organic Synthesis Research Laboratory, Institute of Science, Universiti Teknologi MARA (UiTM) 42300 Bandar Puncak Alam Selangor Malaysia; e Department of Pharmaceutical Technology, Faculty of Pharmacy, Universiti Malaya Kuala Lumpur 50603 Malaysia; f Centre of Foundation Studies UiTM, Universiti Teknologi MARA (UiTM), Cawangan Selangor Kampus Dengkil Dengkil 43800 Malaysia; g Department of Physics, Faculty of Science, Universiti Malaya 50603 Kuala Lumpur Malaysia; h Center for Advanced Materials, Qatar University Doha P.O. Box 2713 Qatar kishor_kumars@yahoo.com; i School of Chemistry, Chemical Engineering and Biotechnology, Nanyang Technological University Singapore 637459 Singapore

## Abstract

The development of electroactive hydrogels as wound dressings represents a promising strategy to actively promote tissue regeneration by providing structural support, electrical stimulation, and localized therapeutic delivery. Poly(3,4-ethylenedioxythiophene):poly(styrenesulfonate) (PEDOT:PSS), a conductive polymer, offers bioelectrical cues *via* its conjugated π-orbitals, but its practical application is limited by instability and leaching under physiological conditions. In this study, we aimed to design and characterize chitosan/gelatin/PEDOT:PSS (CGPP) hydrogels with controlled architecture, mixed ionic–electronic conductivity, and degradability suitable for wound-healing applications. Hydrogels were prepared *via* a cost-effective reverse-casting method using low-melting-point agarose as a sacrificial pore template and were chemically crosslinked for structural stability. Comprehensive characterization, including FESEM, ATR-FTIR spectroscopy, XRD, swelling studies, contact angle measurements, weight loss studies, UV-vis spectroscopy, and electrochemical impedance spectroscopy (EIS), revealed that PEDOT:PSS was successfully integrated into the hydrogel network, producing porous, interconnected architectures with semi-conductive properties (3.78 × 10^−4^ to 2.46 × 10^−3^ S cm^−1^) comparable to native skin tissue. CGPP-4, the formulation with optimal conductivity, exhibited sustained electrical performance over 1 week, was biocompatible, and supported keratinocyte (HaCaT) proliferation and wound closure at biologically relevant concentrations. Incorporation of curcumin further enhanced regenerative outcomes, with 15.625 µg mL^−1^ identified as the optimal dose for complete re-epithelialization. These results highlight the innovative integration of electroconductivity, controlled degradability, and drug delivery in CGPP hydrogels, establishing them as multifunctional platforms for next-generation bioelectronic wound dressings.

## Abstract 2

The development of electroactive hydrogels as wound dressings represents a promising strategy to actively promote tissue regeneration by providing structural support, electrical stimulation, and localized therapeutic delivery.

## Graphical abstract

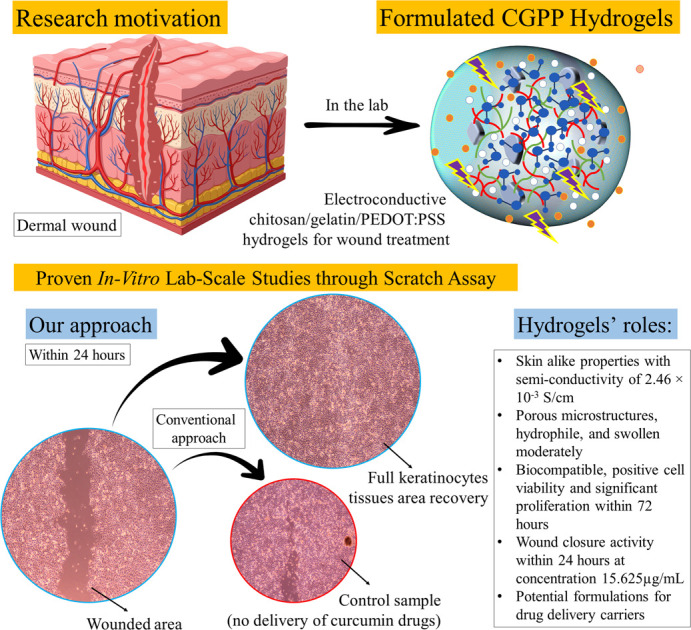

## 1. Introduction

Wound healing treatment, a crucial aspect of the biomedical industry, aims to understand and enhance the natural healing processes of tissues, whether through the use of wound dressings or the application of gels designed to create a suitable environment for tissue healing. Wound healing is a natural biological process that involves the repair of damaged tissues through four main stages: haemostasis, inflammation, proliferation and tissue remodelling.^1^ The field of tissue engineering research is one of the leading branches of innovation in wound-healing technologies to support the recovery process of tissues, specifically in situations where the body's natural healing mechanisms are insufficient.^2^ Currently, a novel form of wound dressing, known as conductive hydrogels, plays an active role in creating a conducive environment for tissues to regenerate through electrical stimulation. Conductive hydrogels have become popular due to their ability to generate electrical signals that enable electrical stimulation in biological systems, regulating cell activities and functions, such as cell proliferation, cell migration, and cell differentiation, which can induce tissue regeneration.^3^ Similar to basic application principles of tissue engineering, an ideal conductive hydrogel for wound treatment should also possess biocompatibility, biodegradability and hydrophilicity that mimic the properties of the extracellular matrix (ECM).^4^ Commonly, hydrogels, hydrophilic 3D polymer network structures, have been formulated using natural or soft polymers like silk, collagen, chitosan, and gelatin to exploit their viscoelastic and porous properties.^4,5^ In order to make the hydrogels electrically conductive, a conductive material such as metal nanoparticles, conducting polymers (CPs), or carbon-based materials was incorporated into the hydrogel mixture through physical or chemical reactions during the fabrication process.^6^ Through our study, we found that our formulated conductive hydrogels demonstrate biocompatibility and are expected to support the natural healing mechanisms, offering a more effective and efficient approach to wound recovery.

According to Zheng *et al.* (2021), tissue regeneration using conductive hydrogels can occur through two distinct mechanisms: active electrical stimulation *via* direct current (DC) and passive support through the hydrogel's intrinsic conductive properties, coupled with endogenous bioelectrochemical signaling.^7^ In the first approach, an external DC power source is applied to the conductive hydrogel, which acts as a bioelectronic interface that delivers controlled electrical stimulation directly to the target tissue. This active method promotes cellular behaviors such as proliferation, migration, and differentiation, especially in excitable tissues like nerves, muscles, and bones, by mimicking or enhancing physiological electric fields. Most studies reported that parameters such as voltage, frequency, and duration can be precisely tuned to optimize regenerative outcomes.^8–11^ In contrast, the second approach does not rely on an external power supply but leverages the hydrogel's inherent conductivity conferred by materials, such as conductive polymers or carbon-based nanomaterials, to support natural cell signaling. This passive method facilitates ion transport and augments native bioelectrical communication within tissues by maintaining or enhancing membrane potentials and intercellular electrochemical signals.^12–14^ While DC stimulation enables rapid and directed regeneration through external control, intrinsic conductive hydrogels contribute to long-term tissue integration by creating an electroactive environment that aligns with the body's natural healing processes. For skin tissue applications, particularly in wound healing and regeneration, electroactive conductive hydrogels with ionic–electronic conductivity are more preferable and suitable than those requiring external DC electrical stimulation.

Conductive hydrogels incorporating conjugated polymers, such as poly(3,4-ethylenedioxythiophene):poly(styrenesulfonate) (PEDOT:PSS), can provide a bioelectrical environment that partially emulates native tissue conductivity. The conjugated polymer backbone facilitates charge transport and can support cellular signaling processes in a manner reminiscent of endogenous bioelectric cues. It should be noted, however, that the electrical properties of skin are highly dependent on measurement frequency, hydration state, and the method employed, and thus any comparison with physiological skin conductivity should be considered as a qualitative rather than absolute benchmark.^6^ Previous studies have also shown that the incorporation of soft and CPs, like polypyrrole, polyaniline, or PEDOT:PSS, into hydrogel systems can activate the electrical conductivity properties in the hydrogel by the movement of charge carriers (electrons or ions) along the CP backbone.^15–17^ Next, this electrical cue provided by conductive hydrogels can enhance cell adhesion and migration at the wound site. This is critical during the initial stages of wound healing, when the cells need to migrate to the wound area for tissue repair. By harnessing the electrical conductivity of these hydrogels, researchers aim to create an environment that enhances cellular activities and supports the various stages of wound healing. While the field is still evolving, early studies and ongoing research suggest promising avenues for the use of conductive hydrogels in advanced wound care applications. This statement is supported by a recent review by Talikowska *et al.* (2019), where they conclude that the application of intrinsically CPs in wound care and skin tissue engineering offers a promising approach to promote faster wound healing, improve antibacterial effectiveness, and enable controlled drug delivery.^18^ The underlying mechanism of its function lies in the fact that human skin's epidermis typically maintains a transepithelial potential, similar to an “internal battery”. When the skin's structural integrity is disrupted, this potential is short-circuited, creating a current at the wound edge. This electrical signal helps guide cells migrate toward the wound center, thereby facilitating the healing process. Additionally, it influences both the direction and rate of cell division.^18^

Among the above-mentioned CPs, PEDOT:PSS has been reported to be widely used in tissue engineering hydrogels because of its simultaneous excellence in conductivity, stability, transparency and biocompatibility.^19^ PEDOT:PSS is composed of conductive π-conjugated PEDOT^+^ and insulating PSS^−^ charged colloidal particles.^6^ To enhance its electrical conductivity, a secondary dopant will be added during the synthesis process. For example, dimethyl sulfoxide (DMSO), a polar solvent, can act as a good secondary dopant to PEDOT:PSS due to its high dipole moment, which can create dipole–charge interactions between PEDOT:PSS and DMSO. This, then, leads to high charge carrier mobility.^6^ However, there is usually an inverse correlation between PEDOT:PSS conductivity and biocompatibility, where the former property can be dramatically increased by secondary doping at the expense of its biocompatibility.^20^ Thus, it becomes essential to conduct thorough cytotoxicity analyses for each PEDOT:PSS-based hydrogel formulation. This ensures that any increase in conductivity does not compromise the hydrogels' suitability for biomedical applications, particularly in tissue engineering. Moreover, based on the published work on conductive hydrogels for wound healing, the therapeutic efficacy and underlying mechanisms of PEDOT:PSS-based hydrogels remain insufficiently characterized and warrant comprehensive investigation. Another concern with the application of PEDOT:PSS-based hydrogels is their instability under physiological conditions due to leaching of doped PEDOT:PSS prior to incubation reactions, leading to a loss of conductivity that compromises the hydrogel's functionality. For example, one study reported that the PEDOT:PSS hydrogel composite remained stable only for 10 minutes before degrading and disintegrating in PBS solution.^21^ Inadequate stability and the use of incompatible hydrogels can disrupt cellular interactions, heightening the risk of harm during tissue regeneration during wound healing.^22,23^ For optimal efficacy in promoting skin tissue recovery through electrical stimulation, it is advisable for formulated hydrogels to acquire conductivity in the range of 2.6 × 10^−3^ to 1.0 × 10^−7^ S cm^−1^, which is on par with natural human skin tissues, ensuring a suitable platform for wound healing applications.^24^

In this study, chitosan/gelatin/PEDOT:PSS (CGPP) conductive hydrogels were designed and systematically investigated as electroactive biomaterials for wound-healing applications. The central aim of the work was to develop a porous, biocompatible hydrogel platform that integrates natural polymers with an intrinsically conductive conjugated polymer and to understand how this hybrid design influences physicochemical behavior, degradation processes, and biological performance relevant to skin regeneration. To achieve this aim, CGPP hydrogels were fabricated using a facile and low-cost reverse-casting strategy combined with chemical crosslinking, in which low-melting-point agarose was employed as a sacrificial template to generate an interconnected porous structure. The study was structured to first characterize the structural and molecular features of the hybrid hydrogels using morphological, spectroscopic, and crystallographic techniques, thereby confirming the successful integration of PEDOT:PSS within the polymer network. This was followed by a systematic evaluation of hydrogel behavior under physiological conditions, focusing on hydrophilicity, swelling, mass loss, and degradation-related changes to assess stability and material evolution over time. Electrochemical analyses were subsequently conducted to examine the conductive behavior of hydrogels during exposure to aqueous physiological environments. Finally, a representative CGPP formulation was selected for biological evaluation to explore the suitability of the electroactive hydrogel as a wound-dressing material. Curcumin was incorporated as a model bioactive compound to assess the feasibility of localized therapeutic delivery, and *in vitro* assays using keratinocyte cultures were employed to examine cytocompatibility and wound-healing-related cellular responses. Through this stepwise approach, the study establishes a comprehensive framework for the rational design and assessment of conductive biopolymer hydrogels aimed at enhancing wound repair.

## 2. Materials and method

### 2.1. Materials

For the fabrication of conductive CGPP hydrogels, a conductive grade of poly(3,4-ethylenedioxythiophene):poly(4-styrenesulfonate) (PEDOT:PSS, 1.3 wt% dispersion in H_2_O), dimethyl sulfoxide (DMSO, >99.9%), acetic acid (>99.7%), chitosan powder (low molecular weight, DD ≥ 70%), gelatin powder (99% pure), agarose powder (low gelling temperature), and glutaraldehyde (25% in H_2_O) were purchased from Sigma Aldrich Corporation (St. Louis, MO, USA). The deionized water used for preparation and lab work was prepared in the lab. For degradation studies and cell work, a phosphate-buffered saline solution (pH 7.4) from Sigma Aldrich was used. Curcumin powder was obtained from Dr Zarif Sofian's laboratory (Faculty of Pharmacy, University of Malaya).

For cell work studies, fetal bovine serum (FBS), Dulbecco modified Eagle's medium (DMEM), and penicillin-streptomycin (pen-strep) were obtained from GIBCO-BRL Life Technologies, New York, USA. Accutase™ in DPBS without Ca/Mg was purchased from Nacalai Tesque Inc., and trypan blue dye for kit cell counting was purchased from Thermo Fisher. Osteoblast-like-osteosarcoma cells MG63, trypsin neutralizing solution and human epidermal keratinocyte cell line HaCaT for wound healing assay were purchased from the American Type Culture Collection (ATCC, Manassas, VA, USA). The remaining chemicals were used as provided by the Department of Pharmaceutical Life Sciences, Faculty of Pharmacy, UiTM Puncak Alam.

### 2.2. Preparation of cs/gel/PEDOT:PSS (CGPP) hydrogels

The PEDOT:PSS aqueous dispersion was enhanced with 3.0 vol% dimethyl sulfoxide (DMSO) to improve electrical conductivity, following a previously reported secondary doping strategy,^6^ with minor modifications as described below. The solution was stirred for 120 min at room temperature to ensure homogeneity. Porous conductive hydrogels were fabricated using a reverse casting and freeze–thaw-assisted gelation approach adapted from established protocols,^25,26^ with minor key modifications. Briefly, a 1.8 wt% chitosan solution prepared in 0.2% (v/v) acetic acid was mixed with 7.2 wt% gelatin at a fixed weight ratio of 1 : 4 under continuous stirring. PEDOT:PSS was incorporated at concentrations ranging from 0 to 6 vol% in 1 vol% increments to systematically tune the electrical properties of the hydrogels. This controlled variation in the conductive polymer content represents an intentional design strategy for optimum conductivity, swelling behavior, and mechanical integrity. To generate a porous architecture, agarose (0.1 g mL^−1^) was introduced as a sacrificial pore-forming template, and the mixture was heated and stirred at 90 °C for 30 min until complete dissolution. Crosslinking was initiated by the addition of 0.5 vol% glutaraldehyde, followed by stirring for 1 min to promote rapid network formation. The pH of the precursor solution was subsequently adjusted to approximately 7.4 to better approximate physiological conditions and improve biocompatibility prior to gelation.^27,28^

**Fig. 1:**
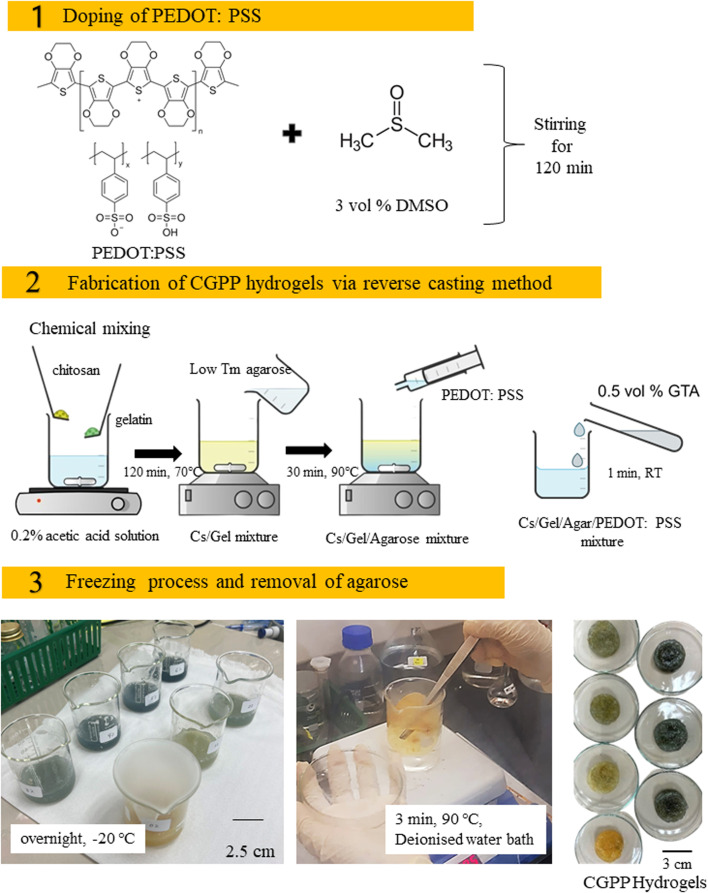
The preparation process of CGPP hydrogels, involving the (1) doping of PEDOT:PSS, (2) its fabrication through the reverse casting method, followed by the (3) freezing of hydrogels and removal of agarose from the hydrogel structure.

The resulting mixtures were cast into cylindrical molds (3 cm diameter) and frozen at −20 °C overnight to induce physical gelation and stabilize the porous structure. After gelation, the frozen hydrogels were thawed and immersed in boiling distilled water (90 °C) for 3 min to selectively remove the agarose sacrificial template, yielding an interconnected porous network. This high-temperature water-immersion step additionally functioned as a post-crosslinking washing process, facilitating the diffusion and hydration of unreacted glutaraldehyde residues, thereby reducing potential cytotoxicity.^29^ Following agarose removal, the hydrogels were equilibrated in phosphate-buffered saline (PBS, pH 7.4) prior to degradation and biological evaluations to ensure stabilization under physiological conditions and minimize residual free aldehyde content. A schematic illustration of the step-by-step CGPP hydrogel fabrication process is provided in Fig. 1. The observed yellowish-to-bluish coloration of CGPP hydrogels correlates with the increasing PEDOT:PSS content, indicating successful dispersion of the conductive polymer within the hydrogel matrix.

### 2.3. Measurements and characterizations of hydrogels

The structural and chemical properties of the hydrogels were evaluated using Field Emission Scanning Electron Microscopy (FESEM), X-ray Diffraction (XRD), and Attenuated Total Reflectance-Fourier Transform Infrared Spectroscopy (ATR-FTIR). For morphological analysis, CGPP hydrogels were mounted on aluminum stubs using carbon adhesive tape and sputter-coated with a thin layer of platinum. The surface and porous architecture were examined using FESEM (ZEISS Supra 40VP, USA) under secondary electron detection in a vacuum. Pore sizes were determined from FESEM micrographs using ImageJ software. Crystallinity was assessed using an X-ray diffractometer (PANalytical X'Pert Pro, Netherlands). The selected hydrogel samples (CGPP-0, CGPP-1, CGPP-3, and CGPP-6) were analyzed to investigate changes in the crystalline structure with increasing PEDOT:PSS content. Prior to measurement, excess surface moisture was removed using tissue paper, and samples were mounted on aluminum stubs with copper tape. XRD patterns were recorded using Cu Kα radiation (*λ* = 1.541 Å) at 45 kV and 40 mA, with a scanning range of 2*θ* = 5–35° and a step size of 0.02°/s. Data were processed using X'Pert Highscore and X'Pert Plus software. The crystallinity index (CrI) was calculated using the Segal peak-height method, which estimates the relative crystalline portion of the material from the XRD pattern.^30^ In this approach, the intensity of the main crystalline peak (*I*_(crystalline)_) and the minimum intensity in the adjacent amorphous region (*I*_(amorphous)_) were extracted from the processed diffractogram in Origin software. The CrI value was then obtained using the equation:
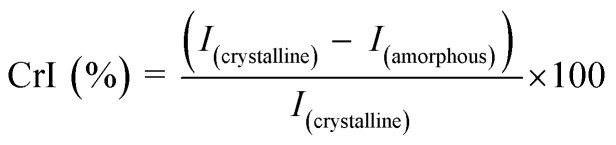


Chemical bonding and functional groups were analyzed using ATR-FTIR (Spectrum, version 10, PerkinElmer, UK). Spectra were collected in the range of 1800–800 cm^−1^ with a resolution of 8 cm^−1^, and four scans were averaged for each sample. Peaks in the spectra were interpreted to identify characteristic vibrational modes of the hydrogel components.

### 2.4. Measurements and characterization techniques of CGPP hydrogels under physiological conditions

For each hydrogel formulation, three independent samples were analyzed (*n* = 3). Data are expressed as mean ± standard deviation, and the standard deviation values are reported in the Results and SI sections for their swelling ratios, weight loss percentage, contact angles and electrochemical conductivity values. The stability of hydrogels in liquid environments is strongly influenced by their swelling behavior. Hydrogels with porous structures can absorb substantial volumes of liquid and swell without dissolving; however, for biomedical applications, excessive swelling is undesirable as it can cause rapid dimensional changes. In this study, the swelling behavior of CGPP hydrogels was assessed using a gravimetric method.^31^ Samples of uniform dimensions were submerged in phosphate-buffered saline (PBS, pH 7.4) at 37 °C. The swelling ratio was recorded at 3 hours intervals from 0 to 30 hours of incubation. Before weighing, hydrogels were rinsed to remove excess surface liquid. The swelling ratio (%) was calculated as follows, where *W*_t_ is the weight after immersion and *W*_o_ is the initial weight.
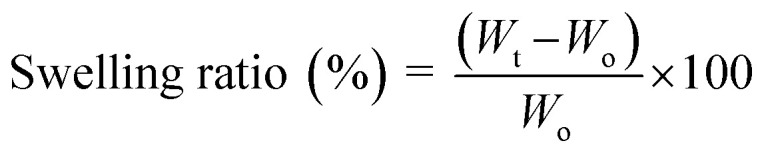


The weight loss and degradation rate of hydrogels in PBS solution were examined over 14 days to evaluate their physical degradation, disintegration and deterioration. The hydrogels were submerged in 10 mL of PBS (pH 7.4) at 37 °C with a hydrogel-to-solution ratio of 1 : 10.^26,32^ At predetermined intervals, samples were removed, rinsed to eliminate residual salts, and weighed. Weight loss (%) was calculated as:
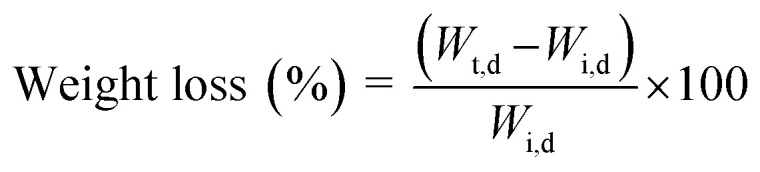


*W*_t,d_ is the dry weight of the hydrogel after incubation for each timepoint, and *W*_i,d_ is the initial dry weight of the hydrogel before incubation. The hydrogels were dried using tissue to estimate the solid content and eliminate the added weight of the buffer. Degradation by-products released into PBS after 14 days were analyzed by UV-vis spectroscopy (Shimadzu UV-Vis 160i, Japan) within the wavelength range 450–750 nm. The concentration of released components was determined using the Beer–Lambert law^33^ where *A* is the measured absorbance, *ε* is the molar absorption coefficient, *b* is the optical path length, and *C* is the concentration.*A* = *εbC*

Functional group analysis of the hydrogels before and after incubation was performed using ATR-FTIR (PerkinElmer Spectrum, version 10, UK). Spectra were recorded in the range of 1800–800 cm^−1^ at 8 cm^−1^ resolution, with four scans averaged for each measurement. Peaks were assigned to specific vibrational modes to assess chemical stability. Hydrophilicity was evaluated using a contact angle goniometer (VCA-3000 s, AST Products Inc., USA) with PBS solution as the test droplet. The contact angle was measured using VCA Optima software, with lower angles indicating higher surface hydrophilicity.

The mixed ionic–electronic conductivity of hydrogels was determined using electrochemical impedance spectroscopy (EIS) with a HIOKI 3520 LCR Hi-Tester over a frequency range of 100–10 000 Hz at room temperature. Conductivity (*σ*) was calculated using following formula^6,14,17,34^ where *L* is the hydrogel length, *R*_b_ is the bulk resistance, and *A* is the cross-sectional area of the electrodes. Two parallel stainless-steel metal electrodes were used, and the hydrogel samples were cut into cylindrical shapes (20 mm diameter, 10 mm length) before being placed between the electrodes in a sandwich configuration. A controlled pressure was applied to ensure good electrical contact without deforming the gels, providing uniform current distribution, minimal interfacial gaps, and a reproducible contact area (*A*). The bulk resistance, *R*_b_ value, is taken from the real axis intercept (*Z*′) of the Nyquist plot at high frequency, where capacitive effects are minimal.

Electrochemical conductivity (ionic):
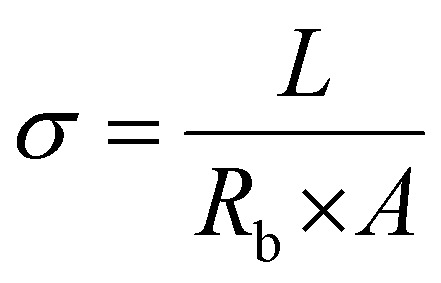


### 2.5. Preliminary toxicity analysis using human osteosarcoma cell line (MG63)

The cytotoxicity and biocompatibility of CGPP hydrogels were evaluated using the human osteosarcoma cell line MG63 as a model due to their robustness, reproducible growth characteristics, and well-established use in early biocompatibility screening. MG-63 cells provide a reliable initial indication of material-induced toxicity, making them suitable for early-stage evaluation before transitioning to more specialized skin-related cell lines. Direct use of primary keratinocytes was not adopted at this stage because they are more sensitive, require stricter culture conditions, and show higher variability, which may complicate initial toxicity screening. The MG63 cells were cultured in Dulbecco's Modified Eagle Medium (DMEM) supplemented with 10% fetal bovine serum (FBS) and 1% penicillin–streptomycin (100 µg mL^−1^ each). Cultures were maintained under sterile conditions in compliance with ISO 10993 at 37 °C in a humidified incubator with 5% CO_2_ and >95% relative humidity. Cells were seeded at a density of 1.5 × 10^5^ cells per well in 12-well plates and incubated for 24 h. The medium was then replaced with fresh DMEM, and the cultures were incubated for an additional 24 h under identical conditions.

Toxicity testing was conducted to ensure that the hydrogel materials did not elicit adverse cellular responses such as inflammation or reduced viability. MG63 cells were treated with CGPP hydrogels for 1 h and 24 h. Following treatment, hydrogels were removed, and cell viability was assessed using an automated cell counter (Invitrogen, Thermo Fisher). Briefly, the culture medium was removed, cells were rinsed with phosphate-buffered saline (PBS), and treated with accutase for 5 min at 37 °C to promote detachment. The cells were then centrifuged at 1100 rpm for 5 min to obtain a pellet. Cell pellets were processed according to the manufacturer's instructions for counting, using Trypan blue exclusion in a countess cell counting chamber slide (Thermo Fisher). Each sample was counted in triplicate. Representative cell images were captured using optical microscopy at 10× magnification. All cytotoxicity experiments were performed in triplicate (*n* = 3) for each CGPP hydrogel formulation. Quantitative cell viability data are reported as mean ± standard deviation, with standard deviation values presented in the Results and SI sections.

### 2.6. Preparations for cell viability and proliferation evaluation of CGPP-4 hydrogels through MTT assay

Curcumin powder was obtained from Dr Zarif Sofian's laboratory (Faculty of Pharmacy, University of Malaya) and incorporated at 1% (w/w) into CGPP-4 hydrogels (cs/gel/PEDOT:PSS 4%). The formulation was stirred for 24 h to ensure homogeneous dispersion. The HaCaT human keratinocyte cell line (passage 59) was used for evaluating cell viability, proliferation, and wound closure performance. Complete DMEM medium was prepared by supplementing 44.5 mL DMEM with 5 mL filtered FBS (10%) and 500 µL penicillin–streptomycin (1%). Cells were thawed from cryogenic storage, centrifuged for 10 min at 5–6 × g (speed), rinsed, and seeded into 23 cm^2^ culture flasks. Once 70–80% confluence was reached, cells were detached with 500 µL trypsin for 5 min at 37 °C, neutralized with fresh medium, and pelleted by centrifugation. Cell counts were determined using a cell counter and microscopy with ToupView software.

For seeding, 96-well plates were prepared with 20 000 cells per well in 100 µL medium. After 24 h of incubation, wells were rinsed three times with PBS. Treatments (CGPP-4 and curcumin-loaded CGPP-4 hydrogels) were prepared by diluting 200 mg mL^−1^ hydrogel in 100% DMSO to obtain 1 mg mL^−1^ stock in 0.5% DMSO–DMEM. Serial two-fold dilutions were prepared to yield final concentrations of 1000, 500, 250, 125, 62.5, 31.25, 15.625, and 7.8125 µg mL^−1^. Each treatment was applied in sextuplicate and incubated for 24 h before viability assessment. The MTT assay was used to assess metabolic activity. Stock MTT solution (5 mg mL^−1^ in PBS, sterile-filtered through a 0.22 µm filter) was diluted to 0.5 mg mL^−1^ in DMEM. After 24 h treatment, 100 µL MTT solution was added to each well and incubated for 3 h at 37 °C. The MTT solution was removed, and formazan crystals were solubilized with 100 µL DMSO. Plates were protected from light and agitated for 10 min before measuring the absorbance at 570 nm (TECAN Infinite 200 PRO). For proliferation assays, the same protocol was applied at 24, 48, and 72 h, using a lower initial cell density than for the MTT assay.^12,20,35^ Each CGPP-4 hydrogel formulation was evaluated using three independent biological replicates (*n* = 3), with each condition tested in sextuplicate wells. Data are presented as mean ± standard deviation, and the corresponding standard deviation values are reported in the results and SI section.

### 2.7. Comparisons of wound healing performance between CGPP-4 and curcumin-loaded CGPP-4 hydrogels through scratch assay

The three most effective hydrogel concentrations from the proliferation assay were selected for scratch assay evaluation:^36^ 31.25, 15.625, and 7.8125 µg mL^−1^. Two treatment groups were tested: (i) CGPP-4 hydrogels, and (ii) curcumin-loaded CGPP-4 hydrogels. According to a previous study, 1% curcumin can improve wound healing through the suppression of inflammatory cytokines.^37^ HaCaT cells were seeded into 6-well plates at 3 × 10^5^ cells per well and cultured until 90% confluence. A linear scratch, as shown in SI 1 (S1), was introduced using a 200 µL pipette tip, followed by washing with 1 mL PBS to remove the debris. PBS was removed, and 2 mL treatment medium was added to each well. Images of the scratch area were captured at 0 h, followed by incubation and subsequent imaging at 18 h and 24 h using an optical microscope. The wound closure area was measured to compare the healing efficacy of hydrogel formulations. All scratch assays were performed using three independent replicates for each hydrogel formulation (*n* = 3). Quantitative wound closure data are expressed as mean ± standard deviation, with standard deviation values presented in the results and SI sections.

## 3. Results and discussions

### 3.1. Effects of PEDOT:PSS incorporation on the morphology, crystallinity and functional group distribution of chitosan/gelatin hydrogels

**Fig. 2:**
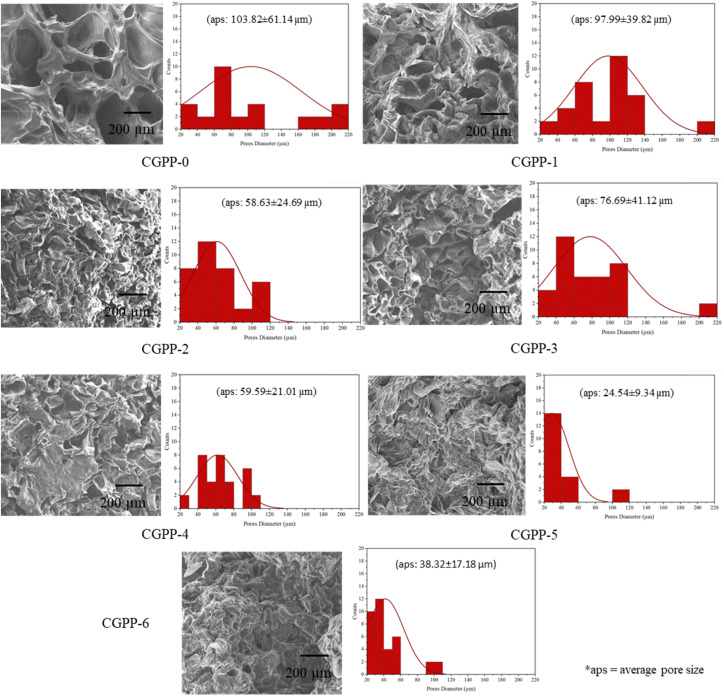
The micrograph of the prepared CGPP hydrogels with a porous interconnected architecture obtained from FESEM analysis, with their respective randomly distributed-average pore sizes measured using Image J software.

To establish the foundational properties of CGPP hydrogels, their structural and compositional characteristics were first examined. Initial analyses focused on fundamental physicochemical attributes, including visual appearance, microstructure, and polymer network organization, to confirm the successful incorporation of PEDOT:PSS within the chitosan–gelatin matrix. These foundational characterizations provide essential context for understanding how hydrogel composition influences porosity, molecular interactions, and material stability, which are critical for subsequent assessments of swelling behavior, degradation, and bioactivity. The characteristic yellowish-blue coloration of CGPP hydrogels corresponds to the varying volume percentages of incorporated PEDOT:PSS, indicating successful dispersion of the conductive polymer within the hydrogel matrix. Morphological examination *via* FESEM revealed that all hydrogel samples exhibited a randomly distributed porous architecture, which is favorable for cellular attachment and nutrient transport, a critical feature for biomedical applications.^38,39^ Quantitative analysis from FESEM images, assisted by Image J software and normal distribution statistics, indicated that the average pore sizes for CGPP-0, CGPP-1, CGPP-2, CGPP-3, CGPP-4, CGPP-5, and CGPP-6 were approximately 103.82 µm, 97.99 µm, 58.63 µm, 76.69 µm, 59.59 µm, 24.54 µm, and 38.32 µm, respectively (Fig. 2). Notably, increasing the PEDOT:PSS content progressively reduced the porosity of hydrogels, while preserving the intrinsic spaces within the polymer network. This phenomenon can be attributed to the long-chain molecular structures of chitosan and gelatin, which have a higher degree of polymerisation when forming hydrogels.^40^ Meanwhile, the semi-crystalline behavior of PEDOT:PSS causes a reduction in pore formation, which might be due to the formation of the S–N bond of sulfenamide between the amino group of chitosan and the thiophene ring of PEDOT, as discussed in our previous findings.^6^ Upon gelation, the hydrogels were immersed in an extraction bath. Synergistically, the reverse-casting fabrication method played a significant role in the generation of pores. Mechanistically, the incorporated low-melting-point agarose in the hydrogel diffused out from the matrix and leaving pore formation due to weak hydrogen bonding formation between the hydroxyl group of agarose and the ether group of chitosan.^6^

**Fig. 3:**
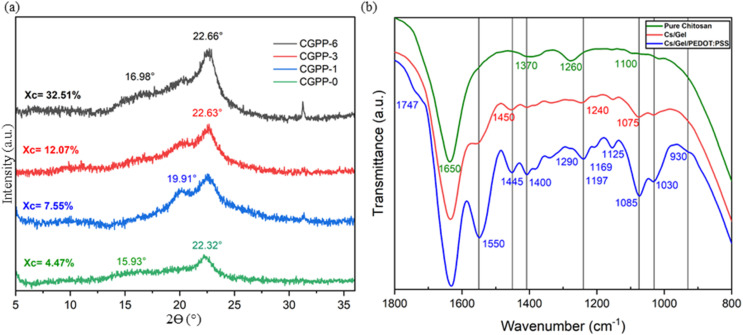
(a) XRD patterns of the CGPP-0, -1, -3 and -6 hydrogels and their respective crystallinity index values, and (b) the ATR-FTIR spectraof the pure chitosan gels, chitosan/gelatin gels at a ratio of 1 : 4 and chitosan/gelatin/PEDOT:PSS hydrogel formulation (CGPP-6).

To assess the crystallinity performance of amorphous CGPP hydrogels, CGPP-0, CGPP-1, CGPP-3, and CGPP-6 with 0, 1, 3, and 6 vol% of PEDOT:PSS content were subjected to XRD analysis in a dried state. As shown in Fig. 3(a), CGPP-0 exhibited a broad diffraction peak intense at 2*θ* ≈ 22.32°, which resembles the characteristic of amorphous chitosan and gelatin. This was supported by findings from Ghosh *et al.*, 2025, who reported that the XRD pattern of pristine chitosan also showed a sharp peak at around 22.0°, consistent with findings from earlier research and indicative of a denser crystalline structure.^41^ One study also revealed that chitosan exhibits a crystalline narrow peak at 2*θ* of 21.7° and an amorphous broad peak at 15.5°, indicating its low crystallinity.^42^ Peak intensity at a range of 20–22.0° also corresponds to triclinic and monoclinic diffraction patterns of gelatin, which can be indicative of semi-crystalline polymeric systems.^43^ The absence of the expected crystalline chitosan peak at 2*θ* ≈ 10.7° likely results from reduced diffraction intensity and the highly hydrated state of the hydrogel matrix.^44^ The strong hydrogen bonding interactions between chitosan and gelatin further support the formation of an amorphous polymer network.^45^

Upon the incorporation of PEDOT:PSS, additional peaks appeared at 2*θ* ≈ 16.98°, 19.91°, possibly due to molecular interactions with the chitosan/gelatin matrix or altered chain packing upon blending.^46,47^ Two characteristic peaks at 17.7° and 25.8° are typically attributed to the amorphous halo of PSS and the interchain packing of PEDOT, respectively.^6^ In this study, the presence of PEDOT:PSS within the hydrogel is evident in the CGPP-0, CGPP-1, CGPP-3 and CGPP-6 samples, which exhibit diffraction features at 16.65° and 22.58°. The slight shifts in peak positions are likely due to structural rearrangements arising from the interaction and integration of the chitosan–gelatin network with the PEDOT:PSS dispersion.^6^ Crystallinity index calculations indicated less significant changes in crystallinity with higher PEDOT:PSS content, ranging from 4.47% to 32.51%. This trend supports the hypothesis that the addition of more amorphous PEDOT:PSS chains might not contribute to the crystallinity of the overall hydrogels. But, it might contribute to the rigid structure formation within the hydrogel network because of the formation of more S–N bonds in sulfenamide, a strong and stable covalent bond.^48^

ATR-FTIR analysis (Fig. 3b) further confirmed the molecular composition of chitosan gels, chitosan/gelatin gels and CGPP-6 hydrogels. Distinct absorption bands corresponding to chitosan were observed at ∼1100 cm^−1^ (C–O–C symmetric stretching), ∼1370–1380 cm^−1^ (C–H bending and C–O stretching), and 1654 cm^−1^ (C=O stretching), along with a broad N–H band near ∼1650 cm^−1^ and an acetyl group peak at ∼1260 cm^−1^, indicating partial retention of chitin-like units. Gelatin incorporation introduced new peaks at ∼1075, ∼1240, and ∼1450 cm^−1^, attributed to C–O and C–H stretching, N–H bending (amide III), and C–H deformations, respectively.^6,49,50^ For successful PEDOT:PSS incorporation in CGPP hydrogels, the absorption bands at 1030 and 1400 cm^−1^ correspond to S–O symmetric stretching and CH_3_ groups, respectively, originating from DMSO, which is used as a secondary dopant to enhance the conductivity of PEDOT. A notable peak at 1747 cm^−1^ corresponds to the doped state of PEDOT, confirming its integration into the hydrogel network.^4,6,51^ The characteristic PEDOT:PSS peaks at 930 cm^−1^ (C–S), 1085 cm^−1^ (S–phenyl), 1197 cm^−1^ (S–O), and ∼1400 cm^−1^ (C–O–C) indicate the presence of PEDOT:PSS within the hydrogel matrix. The expected PEDOT:PSS peak at 1523 cm^−1^ (C–C) appears shifted to ∼1445 cm^−1^, likely due to strong spectral overlap with the dominant gelatin and chitosan bands. Similarly, the typical C=C stretching peak around 1550–1624 cm^−1^ is masked or shifted as a result of interference from the polymeric backbone of chitosan and gelatin.^52^

### 3.2. Properties of CGPP hydrogels under physiological conditions

#### 3.2.1. Swelling behaviour, biodegradation and leached species correlation of CGPP hydrogels

**Fig. 4:**
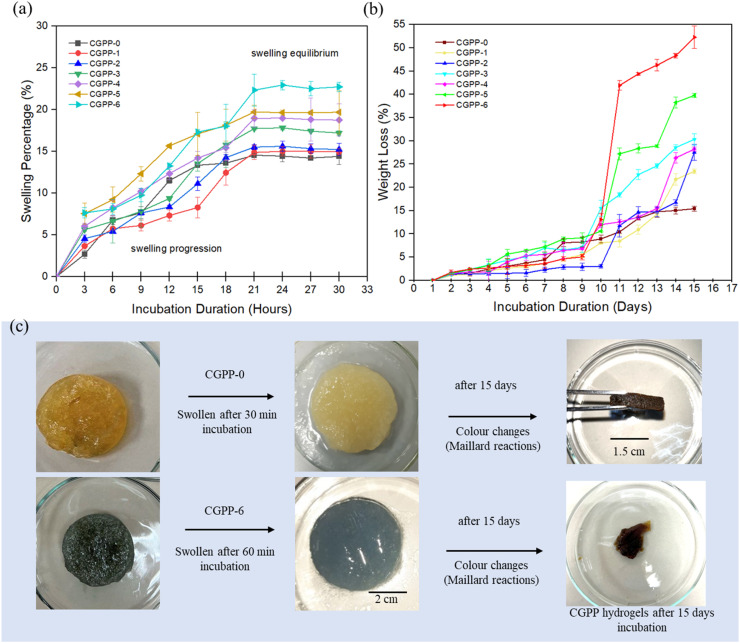
(a) The swelling behaviour trend of the CGPP hydrogels within 30 hours incubation under physiological conditions showing most of the hydrogels started reaching swelling equilibrium at hour-21, (b) the weight loss percentage of the CGPP hydrogels measured within 15 days of incubation showing degrading behaviour, and (c) images of the swollen CGPP-0 and CGPP-6 hydrogels after 60 minutes of incubation (left) and images of the CGPP-2, CGPP-6 degraded hydrogels, and other CGPP hydrogels after 15 days of incubation, experiencing brownish-colour changes (right).

The swelling behavior of CGPP hydrogels was monitored over a 30 hours period, with weight measurements recorded every 3 hours during incubation. All hydrogel formulations demonstrated a typical swelling profile, reaching equilibrium after approximately 21 hours (Fig. 4a). A shorter time to reach equilibrium reflects a more uniform internal microstructure and efficient water penetration throughout the hydrogel matrix.^24^ Among all samples, the CGPP-6 hydrogel exhibited the highest swelling percentage (22.34%) at 21 hours, whereas the CGPP-0 hydrogel, despite its faster initial swelling (3–15 hours), plateaued at a significantly lower swelling percentage (14.55%). This contrast highlights the role of PEDOT:PSS in water uptake. Specifically, the polystyrene sulfonate (PSS) moiety in PEDOT:PSS possesses hygroscopic properties, contributing to increased hydrophilicity and swelling capacity. Visual observations also confirmed that CGPP-6 retained more water than CGPP-0 after 60 minutes under physiological conditions (Fig. 4c), suggesting that increasing the PEDOT:PSS content improves hydrogel swelling behavior through enhanced moisture affinity.

Weight-loss analysis further confirmed the hydrogel's degradability under physiological conditions (PBS, pH 7.4). All CGPP formulations exhibited varying biodegradation profiles over a 2 weeks incubation period (Fig. 4b). Notably, CGPP-6 demonstrated the highest weight loss percentage (52.27%), followed by CGPP-5 (39.78%), while CGPP-0, -1, and -2 showed more gradual and limited degradation. This trend is quite similar to reported research on chitosan/gelatin hydrogel scaffolds, where we can see that by increasing the immersion time, degradation continued with a significant loss of mass.^53^ Moreover, our recorded weight loss of hydrogels aligns with the swelling data, suggesting that increased water uptake and retention facilitate greater material disintegration and mass loss. The calculated degradation rates summarized in SI 2 (S2) support this conclusion. This *in vitro* behaviour of hydrogels was also proposed in the later part of the results section. CGPP-6, with the highest PEDOT:PSS content (6 vol%), exhibited the fastest degradation rate at 0.0026 g min^−1^, while CGPP-0 displayed the slowest rate (0.0008 g min^−1^). Some hydrogels began to lose structural integrity after 15 days, indicating a functional mechanical lifespan of approximately two weeks under physiological conditions (Fig. 4c). Some studies reported that the chitosan/gelatin network is likely to undergo enzymatic degradation instead of hydrolytic degradation. But we believe that there are weak ionic interactions between NH_3_^+^ from chitosan and COO^−^ from gelatin, which cause disruption in the hydrogel matrix to some degree and can supersede crosslinking efforts.^6^

**Fig. 5:**
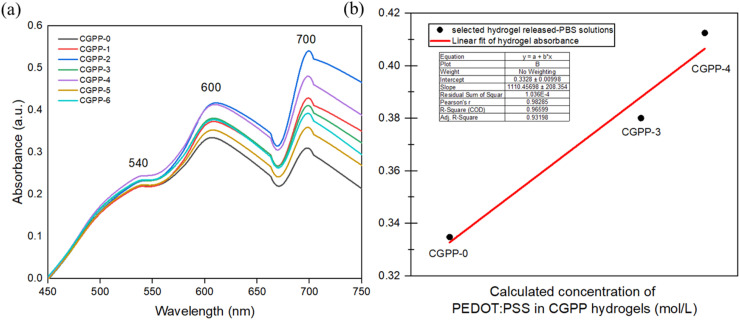
(a) UV-vis spectra of PBS incubation media used for the CGPP hydrogel degradation studies, showing qualitative absorbance features associated with leached species released under physiological conditions. (b) Linear regression analysis of UV-vis absorbance intensity in PBS, illustrating the relative extent of material release from the CGPP hydrogels during degradation; the detected leachates represent a mixture of biopolymer-derived fragments and minor PEDOT:PSS-related species rather than fully identified individual components.

Further analysis was performed to determine the nature of the hydrogel components released into PBS during incubation. Because all hydrogel samples exhibited degradation and measurable weight loss under physiological conditions (37 °C, pH 7.4), the PBS supernatant was collected at specific time points and subjected to UV-vis spectroscopy. This approach allowed us to identify and monitor the species liberated from the hydrogel matrix throughout the degradation process. As plotted in Fig. 5a, distinct absorption peaks at ∼540, ∼600, and ∼700 nm were detected in the PBS solution, particularly from CGPP-2 and CGPP-4 hydrogels. These peaks are characteristic of π–π* transitions within the thiophene rings of PEDOT:PSS, as previously reported,^54,55^ suggesting that some leaching of PEDOT:PSS occurred. Interestingly, CGPP-0 hydrogel (composed only of crosslinked chitosan and gelatin) also exhibited broad absorbance in the 500–800 nm range, which is consistent with reported gelatin and chitosan degradation signatures.^56^ Notably, we believe the hydrogels are highly miscible and form an interconnected network in which PEDOT:PSS, chitosan, and gelatin are uniformly distributed. This cohesive structure likely promotes collective physical deterioration of the hydrogel as a bulk material rather than the release of isolated components. In other words, instead of PEDOT:PSS diffusing independently out of the polymer matrix, the entire network gradually erodes together, reflecting the strong physical and chemical integration achieved in the fabricated gels.

Moreover, our hydrogels exhibited a visible color change from yellow-bluish to brownish after several days of incubation, particularly in PEDOT:PSS-containing samples (Fig. 4c). This browning likely arises from a combination of partial leaching of PEDOT:PSS components and Maillard-type reactions between gelatin and chitosan-derived degradation products.^57^ The Maillard reaction is a non-enzymatic browning process involving the condensation of free amino groups (lysine residues in gelatin or protonated amines from chitosan) with carbonyl-containing degradation products from polysaccharides, leading to the formation of melanoidins and other complex, crosslinked structures. These reactions contribute to the observed color change and may influence the microstructural stability of the hydrogel matrix over time.^58^ UV-vis analysis of the PBS incubation medium revealed the presence of leached species, although the specific chemical identities could not be unambiguously determined with this method. Based on the spectral features, we infer that the released materials likely comprise a mixture of small biopolymer fragments (chitosan and gelatin) and minor PEDOT:PSS oligomers or unbound fragments, consistent with previous studies on conductive hydrogel leaching. Quantitative analysis (SI 3, S3) and linear regression of selected samples (Fig. 5b) indicate that CGPP-2 and CGPP-4 released more material into the medium, possibly due to increased matrix porosity or local phase separation associated with PEDOT:PSS incorporation.

Despite this partial leaching, hydrogels retained over 75 wt% of their initial mass after 14 days (weight-loss analysis), highlighting their suitability for short-term therapeutic applications, such as minor wound healing, where tissue repair typically occurs within one week. PEDOT:PSS, while generally hydrolytically stable, may partially diffuse from the hydrophilic hydrogel matrix due to water uptake and ionic interactions, especially involving the PSS component. Interactions between the protonated chitosan amino groups (–NH_3_^+^) and the π-electron-rich thiophene rings of PEDOT (cation–π interactions) enhance polymer compatibility and contribute to a more interconnected, compact hydrogel network.^59^ This network organization reduces free volume and restricts water penetration, slowing hydrogel swelling (Fig. 4a) and limiting independent PEDOT:PSS diffusion.

Glutaraldehyde crosslinking also stabilizes the hydrogel network while leaving residual aldehyde groups that may facilitate slow structural reorganization or degradation. Overall, the UV-vis findings suggest a dynamic, mixed-mode degradation profile, governed by covalent crosslinking, ionic interactions in PBS, secondary reactions such as Maillard browning, and partial leaching of PEDOT:PSS and biopolymer fragments. The observed leachates are expected to be largely biocompatible, given the known cytocompatibility of this hydrogel under physiological conditions (discussed in Section 3 – Biocompatibility of CGPP hydrogels, proliferation and wound healing properties of CGPP-4 hydrogels through delivery of curcumin drug), supporting the potential of hydrogels for wound-healing applications.

#### 3.2.2. Chemical structural changes, hydrophilicity and electrochemical functionality of CGPP hydrogels after degradation

To investigate the chemical stability and degradation pattern of the hydrogels, ATR-FTIR was performed after 15 days of incubation in phosphate-buffered saline (PBS) and contact angle analyses were conducted on the hydrogel samples. Hydrogels typically degrade *via* two mechanisms: bulk degradation, which occurs throughout the matrix, and surface degradation, which is limited to the exterior.^4^ The hydrogels in this study exhibited signs of bulk degradation early during incubation, likely due to their high swelling behavior and internal structural disintegration. Specifically, CGPP-6, which contains the highest proportion of PEDOT:PSS, demonstrated the poorest mechanical integrity and dimensional stability, which correlated with its highest swelling and weight-loss percentages. The increase in PEDOT:PSS content corresponded to a rise in swelling capacity, which could disrupt crosslinking bridges and accelerate the hydrolytic degradation process. Additionally, the presence of pores in the hydrogel matrix may have further contributed to the diffusion of water and degradation kinetics.

**Fig. 6:**
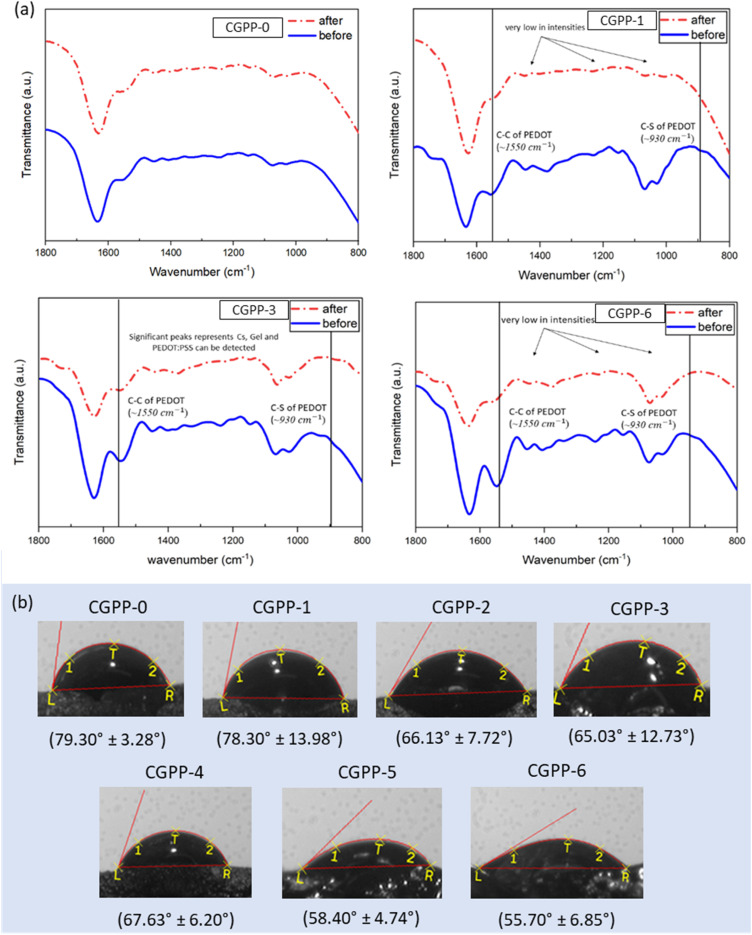
(a) The ATR-FTIR plot of the CGPP-0, CGPP-1, CGPP-3 and CGPP-6 hydrogels (taken before and after incubation in physiological conditions) and (b) the surface wettability of hydrogels upon contact with the PBS solution from contact angle measurements.

ATR-FTIR spectra of the remaining hydrogels (Fig. 6a) revealed characteristic peaks for chitosan and gelatin in CGPP-0, -1, -3, and -6 after incubation, indicating partial retention of these natural biopolymers. However, PEDOT:PSS-associated peaks appeared diminished, especially in CGPP-1, where the overall spectrum was significantly flattened, suggesting substantial material loss. In contrast, CGPP-3 and CGPP-6 retained identifiable PEDOT:PSS peaks, particularly the C–S bond (∼930 cm^−1^) and C–C skeletal vibrations in the thiophene ring (∼1550 cm^−1^),^6,51^ confirming that higher PEDOT:PSS loading improves its retention during degradation. Nevertheless, signals within the 1100–1300 cm^−1^ range, typically associated with the PEDOT:PSS sulfonate vibrations, were notably weaker, possibly due to leaching or structural rearrangement. UV-vis spectroscopy of the PBS solution post-incubation further supported the release of hydrogel components into the medium. The observed absorbance in the visible range (540, 600, and 700 nm) corresponds to conjugated systems and chromophores that may originate from aromatic groups or degradation by-products, indicating active solubilization of hydrogel constituents over time.

The enhanced degradation of highly swollen hydrogels may result from a lower crosslinking density due to the hydrophilic and bulky nature of PSS, which disrupts network tightness. Consequently, water penetrates more easily, accelerating hydrolysis and the diffusion of polymer chains, particularly chitosan and gelatin, out of the matrix. Interestingly, while gelatin is traditionally reported to exhibit hydrophobic characteristics in film and hydrogel forms,^60,61^ contact angle measurements revealed that PEDOT:PSS incorporation significantly improved surface wettability of CGPP-hydrogels (Fig. 6b). Moreover, we confirmed an increase in the hydrophilicity of hydrogels, as evidenced by a decrease in the measured contact angle from 79.27° ± 6.75° in CGPP-0 to 55.73° ± 7.63° in CGPP-6 due to the hygroscopic nature of PSS for water uptake. This improvement is attributed to the abundance of negatively charged sulfate groups from the PSS chains, which interact favorably with the aqueous environment, allowing the PBS droplet to spread more readily on the hydrogel surface.^62^ This increase in hydrophilicity, combined with the swelling and degradation behavior, supports the suitability of CGPP hydrogels for biomedical applications. Enhanced surface wettability is beneficial for cell adhesion, proliferation, and overall bioactivity, underscoring the potential of chitosan/gelatin-based PEDOT:PSS hydrogels as bio-interactive wound dressings. Importantly, the release of small biopolymer fragments from the hydrogels during degradation is expected to be inherently biocompatible and may even provide positive cues for cell proliferation and wound-healing processes. Chitosan and gelatin fragments can serve as substrates for cellular attachment or as signaling molecules that stimulate regenerative responses, while the amount of PEDOT:PSS leached into the medium was minimal and dispersed, indicating a low risk of cytotoxic effects.^63,64^

**Fig. 7:**
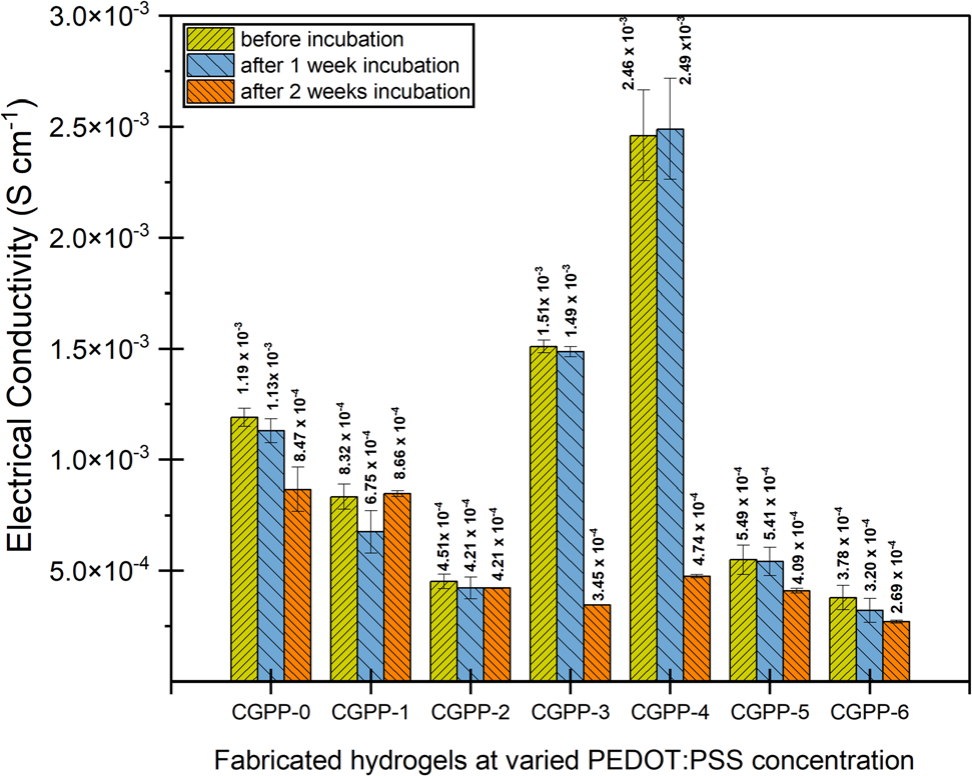
Effective mixed ionic–electronic conductivity behaviour of the CGPP hydrogels before incubation and after 1 and 2 weeks of incubation under physiological conditions, as measured by electrochemical impedance spectroscopy (EIS).

To further understand the structural and electrochemical stability of CGPP hydrogels after prolonged exposure to physiological conditions, electrochemical impedance spectroscopy (EIS) measurements were performed on hydrogel samples after incubation. This characterization provides insight into changes in charge-transport behaviour induced by hydrolytic degradation within the hydrated polymer network.^65^ EIS measurements were conducted on the incubated hydrogels to assess their electrical stability before and after degradation (Fig. 7 and SI 4 (S4)). For wound-healing applications, hydrogels with electroactive properties are desirable, as electrical cues have been shown to support cellular signalling, migration, alignment, and proliferation by mimicking native electrophysiological environments.^66^ In the present study, EIS measurements were carried out on fully hydrated, PBS-equilibrated samples; therefore, the reported conductivity values represent effective mixed ionic–electronic conductivity, rather than purely electronic transport.

**Fig. 8:**
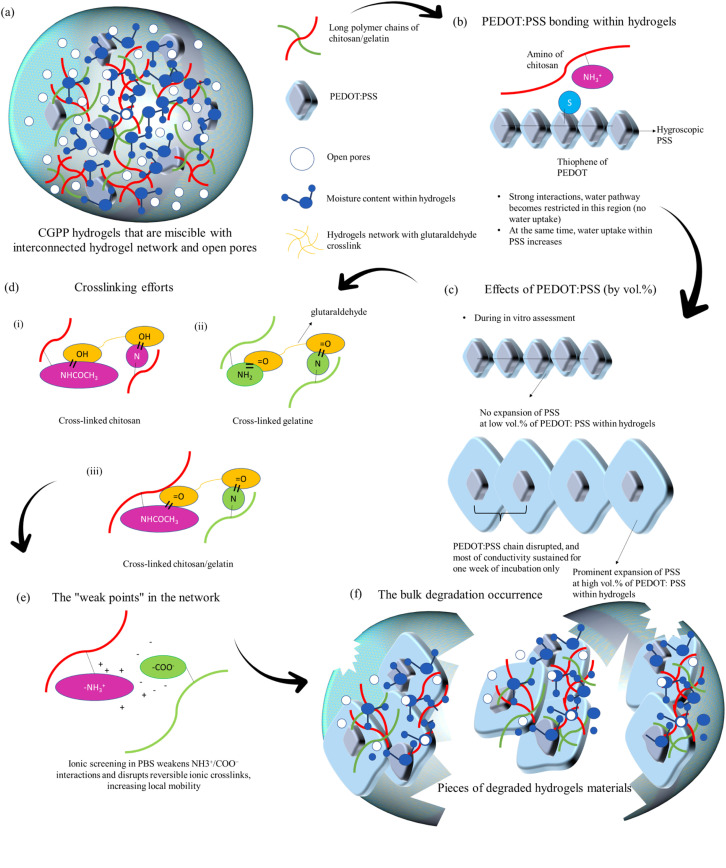
Schematic of the proposed and hypothesized mechanisms of degradation and structural evolution of chitosan/gelatin/PEDOT:PSS (CGPP) hydrogels during incubation in PBS (37 °C, pH 7.4). (a) Formation of an interpenetrating, miscible hydrogel network comprising glutaraldehyde-crosslinked chitosan (red) and gelatin (green), with uniformly distributed PEDOT:PSS domains (blue, with PSS chains) and reverse-cast porous architecture. (b) and (c) Development of heterogeneous microdomains upon hydration, where hydrophilic PSS-rich regions undergo pronounced swelling, while PEDOT–chitosan-associated domains (stabilized by non-covalent interactions such as cation–π and electrostatic associations) resist water penetration, generating internal mechanical stress. (d) Covalent crosslinking within the chitosan–gelatin network provides the primary structural integrity. (e) PBS infiltration and ionic screening of reversible NH_3_^+^/COO^−^ interactions promote early-stage swelling (equilibrium at approximately 21 h). (f) Progressive swelling, partial PSS leaching, and increased PEDOT inter-domain spacing disrupt mixed ionic–electronic percolation pathways, leading to a gradual decrease in conductivity (EIS) and bulk erosion behaviour consistent with observed weight loss and colour changes.

CGPP-0 exhibited a relatively high conductivity (1.19 × 10^−3^ S cm^−1^), which is primarily attributed to ionic conduction originating from chitosan, a cationic polyelectrolyte containing protonated amino groups that facilitate ion transport in aqueous media. Upon the incorporation of 1 vol% PEDOT:PSS (CGPP-1), a slight reduction in conductivity was observed, likely due to partial disruption of established ionic pathways or insufficient formation of interconnected PEDOT:PSS domains at low loading. In contrast, hydrogels containing 3–4 vol% PEDOT:PSS (CGPP-3 and CGPP-4) showed a pronounced increase in conductivity, with CGPP-4 exhibiting the highest value. This enhancement suggests the formation of more continuous PEDOT:PSS domains that increase the electronic contribution within the overall mixed-conducting system. At higher PEDOT:PSS loadings (CGPP-5 and CGPP-6), conductivity decreased, potentially due to aggregation or saturation effects that limit efficient charge transport.^67^ After 7 and 14 days of PBS incubation, a general decline in conductivity was observed across all hydrogel samples, which can be attributed to the leaching of mobile ions and partial degradation of conductive components. In PEDOT:PSS-containing hydrogels, this behaviour may be further influenced by the hydrophilic nature of the PSS counterion, which absorbs water and increases inter-domain spacing between PEDOT-rich regions, thereby reducing both ionic and electronic transport efficiency^68^ (as illustrated in Fig. 8). As both chitosan and PSS are highly hydrophilic, prolonged interaction with PBS promotes water uptake and structural rearrangements within the hydrogel matrix, affecting charge-transport pathways.

Notably, CGPP-4 maintained relatively stable conductivity after one week of incubation, indicating good structural integrity and sustained electroactivity under physiological conditions. The slight increase observed at this stage may be associated with the redistribution or enhanced mobility of residual PBS ions within the hydrogel network. After two weeks of incubation, the conductivity of CGPP-4 decreased by approximately one order of magnitude, but remained within the semiconductive range (4.74 × 10^−4^ S cm^−1^). This sustained mixed ionic–electronic conductivity is a key characteristic for bioelectronic wound dressings, where electrical cues are harnessed to modulate cellular behaviour and enhance tissue regeneration.

#### 3.2.3. Proposed degradation mechanisms of CGPP hydrogels

Our combined FESEM, FTIR, swelling, weight-loss, contact-angle, and EIS results collectively indicate that chitosan, gelatin, and PEDOT:PSS form a miscible and interconnected hydrogel network with open porosity generated *via* the reverse-casting method. The long polymer chains of chitosan and gelatin establish a highly entangled backbone with a high degree of polymerisation, while glutaraldehyde crosslinking introduces covalent stabilization through Schiff base and hemi-acetal type linkages between amino and carbonyl groups, thereby reinforcing the bulk network structure. PEDOT:PSS is uniformly distributed within this matrix and contributes both hydrophilic (PSS) and π-conjugated (PEDOT) domains, as supported by characteristic FTIR bands associated with C–S, S–phenyl, S–O, and thiophene skeletal vibrations. Based on the collective data, three principal types of interactions are proposed to govern the structure and degradation behaviour of CGPP hydrogels (as illustrated in Fig. 8). First, covalent crosslinks introduced by glutaraldehyde provide primary structural integrity and remain relatively stable under neutral PBS conditions.^69^ Second, ionic interactions between protonated chitosan amino groups (–NH_3_^+^) and gelatin carboxylates (–COO^−^) act as weaker, reversible crosslinks that are susceptible to ionic screening, forming localized domains that function as mechanically vulnerable regions within the network.^70^ Third, interactions between PEDOT:PSS and chitosan are proposed to arise primarily from non-covalent mechanisms, including cation–π interactions between protonated chitosan amines and the π-electron-rich thiophene rings of PEDOT,^71^ as well as electrostatic interactions involving the sulfonate groups of PSS. Possible sulfenamide-like S–N interactions are hypothesized based on FTIR peak shifts and attenuated thiophene-related bands following incubation; however, these observations are interpreted as indicative of changes in the local chemical environment rather than definitive evidence of covalent bond formation. The FTIR and EIS trends collectively support the presence of such associative interactions, which enhance component compatibility and promote compact PEDOT-rich microdomains within the hydrogel matrix.

Upon immersion in PBS, the hygroscopic PSS component absorbs water, increasing local free volume and promoting swelling. This behaviour is consistent with the higher swelling ratios observed for CGPP-6, reduced contact angles, enhanced wettability with increasing PEDOT:PSS content, and visible water retention. Regions where PEDOT–chitosan interactions are more compact exhibit reduced local water uptake, likely due to the exclusion of water molecules from tightly associated cation–π domains.^72^ Consequently, the hydrogel exhibits heterogeneous swelling behaviour, with PSS-rich regions swelling significantly while PEDOT–chitosan-rich domains resisting expansion, generating internal mechanical stress.^73^ During the early stage of incubation, rapid water uptake into the PSS-rich pores is observed, with swelling approaching equilibrium at approximately 21 h. Simultaneously, ionic screening in PBS weakens the reversible –NH_3_^+^/−COO^−^ interactions, increasing polymer chain mobility.^74^ FTIR spectra retain most polymer signatures with minor shifts and attenuations, while EIS responses vary depending on PEDOT:PSS loading, reflecting the balance between ionic transport and evolving mixed-conductive pathways. At the intermediate stage, continued swelling enhances hydrolytic access to covalent crosslinks as well as glycosidic and peptide bonds, leading to increased mass loss (CGPP-6 > CGPP-5 > CGPP-0). UV-vis spectra of PBS reveal leached chromophores associated with PEDOT:PSS π–π* transitions and broader polymer degradation products. FTIR analysis of residual hydrogels shows attenuation of PSS sulfonate bands (1100–1300 cm^−1^), suggesting partial leaching or structural rearrangement. Concurrently, EIS conductivity decreases as PEDOT inter-grain connectivity is disrupted by water uptake and PSS swelling, which increases the inter-domain spacing (as illustrated in Fig. 8).

At the final stage of degradation, bulk structural deterioration becomes evident, with localized collapse of the hydrogel network rather than selective dissolution of individual components (Fig. 8). This behaviour correlates with observed browning associated with Maillard-type reactions between the amino groups and carbonyl-containing degradation products, as well as a marked loss of mechanical integrity. Residual fragments are likely stabilized by the remaining covalent crosslinks and more compact PEDOT–chitosan-associated regions. The high miscibility and uniform distribution of PEDOT:PSS within the chitosan/gelatin matrix favour collective erosion of the hydrogel, as PEDOT:PSS is physically interpenetrated within the polymer network and further associated through non-covalent interactions.^75^ Consequently, the system degrades as an ensemble under swelling and ionic attack rather than releasing isolated PEDOT chains alone. While UV-vis analysis confirms some leaching of PEDOT-related species, the concurrent release of chitosan and gelatin degradation products indicates bulk degradation. Swelling of PSS increases the PEDOT–PEDOT inter-domain distances, and partial leaching disrupts continuous mixed-conductive pathways, consistent with the time-dependent decrease in EIS conductivity. The proposed mechanisms of degradation and physical deterioration of CGPP hydrogels are schematically illustrated in Fig. 8.

### 3.3. Biocompatibility of CGPP hydrogels, proliferation and wound healing properties of CGPP-4 hydrogels through the delivery of curcumin drug

#### 3.3.1. Cytocompatibility of CGPP hydrogels as a preliminary analysis of cell viability

In this study, the cytocompatibility of CGPP hydrogels containing varying concentrations of PEDOT:PSS (0–6 vol%) was systematically evaluated to assess their safety profile and suitability for biomedical applications. Particular attention was given to potential cytotoxic effects associated with residual crosslinking agents, as glutaraldehyde was employed during hydrogel fabrication. As described in the Methods section, the hydrogels underwent high-temperature aqueous washing during agarose template removal, followed by prolonged PBS equilibration, which together are expected to effectively reduce free aldehyde residues prior to biological testing.^76^ Moreover, the release of small biopolymer fragments from the hydrogels during degradation is expected to be inherently biocompatible and may even provide positive cues for cell proliferation and wound-healing processes, as described previously in the degradation section. This controlled and limited leaching suggests that the conductive component remains largely embedded within the hydrogel matrix, maintaining its bioelectronic functionality while avoiding potential adverse effects from free conductive polymers.

**Fig. 9:**
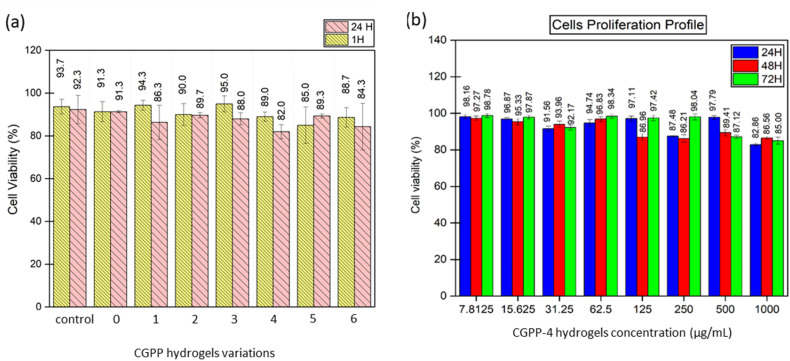
(a) The cell viability plot of osteosarcoma MG-63 cells at 1- and 24 hours incubation time across CGPP hydrogel variations and (b) the proliferation profile of keratinocyte HaCaT cells at 24-, 48- and 72 hours incubation upon the CGPP-4 hydrogel formulation.

Preliminary toxicity screening was conducted using MG63 human osteosarcoma cells, a commonly employed *in vitro* model for assessing material cytocompatibility. Trypan blue exclusion assays showed that all CGPP formulations maintained cell viabilities above 80% after both 1 h and 24 h of exposure, indicating the absence of acute cytotoxic effects across all compositions^77^ (Fig. 9a). Notably, CGPP-1 and CGPP-3 (containing 1 and 3 vol% PEDOT:PSS, respectively) exhibited the highest viabilities after 1 h, while CGPP-5 displayed a modest increase in viability from 85.0% to 89.3% after 24 h, suggesting favorable cell tolerance over time.

CGPP-0 (PEDOT:PSS-free hydrogel) showed relatively unchanged viability over 24 h, whereas PEDOT:PSS-containing hydrogels exhibited slight viability reductions that nevertheless remained well within accepted biocompatibility thresholds. This trend likely reflects cellular adaptation to differences in surface chemistry and charge distribution rather than a toxic response.^78^ Importantly, the sustained cell viability observed across all formulations indicates that residual aldehyde content following post-treatment was below biologically harmful levels. Optical microscopy further confirmed cytocompatibility, with no evidence of abnormal morphology, cell detachment, or membrane damage, and with viable cell clustering observed on hydrogel surfaces (SI Fig. S5). These findings are consistent with prior reports demonstrating that PEDOT:PSS-based biomaterials can support cell adhesion and proliferation when appropriately processed.^79,80^ The cytocompatibility results support the effectiveness of the implemented post-crosslinking washing strategy and confirm that CGPP hydrogels, particularly those containing low to moderate PEDOT:PSS contents are non-toxic and suitable for subsequent keratinocyte-based wound-healing studies. The system therefore provides a safe and biofunctional platform that integrates structural support with mixed ionic–electronic properties relevant to regenerative applications.

#### 3.3.2. The effects of CGPP-4 hydrogels on the proliferation of keratinocyte HaCaT cells

The CGPP-4 hydrogel formulation, incorporating 4 wt% PEDOT:PSS within a chitosan-gelatin matrix, was selected for *in vitro* biological evaluation based on its physiological-level electrical conductivity and structural stability under aqueous conditions. The measured conductivity of CGPP-4 was within the range reported for native human skin tissues^81,82^ and electrochemical impedance spectroscopy (EIS) confirmed that the hydrogel retained measurable conductivity after one week of incubation in PBS. A modest change in impedance during this period may be attributed to ion redistribution within the hydrated network. After two weeks of incubation, the conductivity decreased by approximately one order of magnitude but remained within the semiconductive range (4.74 × 10^−4^ S cm^−1^), indicating partial disruption of conductive pathways while preserving overall network integrity.^83^

Rather than attributing biological effects solely to conductivity, CGPP-4 was considered a multifunctional material system in which electrical properties coexist with favorable surface chemistry, swelling behavior, porosity, and polymer composition. To evaluate cytocompatibility, human keratinocyte (HaCaT) cells were exposed to CGPP-4 at concentrations ranging from 7.8125 to 1000 µg mL^−1^, alongside positive (culture medium) and negative (DMSO) controls. As shown in Fig. 6b, high cell viability (>90%) was observed at lower concentrations (7.8125–62.5 µg mL^−1^) across 24, 48, and 72 h, indicating that CGPP-4 supports keratinocyte proliferation without inducing cytotoxic effects (Fig. 9b). At higher concentrations, particularly 1000 µg mL^−1^, a dose-dependent reduction in viability was noted, which may be associated with osmotic effects or local accumulation of charged polymeric species.^84^ Optical microscopy images acquired after 72 h (SI 6, S6) revealed normal HaCaT morphology at biologically relevant concentrations, further confirming the cytocompatibility of CGPP-4. Collectively, these results indicate that CGPP-4 provides a cell-supportive environment, where electrical conductivity may act as a contributing factor alongside material wettability, swelling capacity, and ECM-mimetic composition. Based on these findings, concentrations of 7.8125, 15.625, and 31.25 µg mL^−1^ were selected for subsequent scratch-wound assays to examine keratinocyte migration.

From a mechanistic perspective, the incorporation of CGPP-4 within keratinocyte cultures is primarily governed by physical and electrochemical interactions between the hydrated polymeric network and the cell membrane, rather than by the formation of new covalent bonds. The chitosan–gelatin matrix provides an ECM-mimetic environment rich in polar functional groups (–NH_2_, –OH, and –COOH), which facilitates cell adhesion through hydrogen bonding, electrostatic interactions, and protein-mediated anchoring at the cell–material interface.^85^ The porous architecture further enables partial cell infiltration and close cell-hydrogel contact, promoting efficient ion exchange within the hydrated network.

#### 3.3.3. Wound healing performance of keratinocyte HaCaT tissue with CGPP-4 hydrogels

**Fig. 10:**
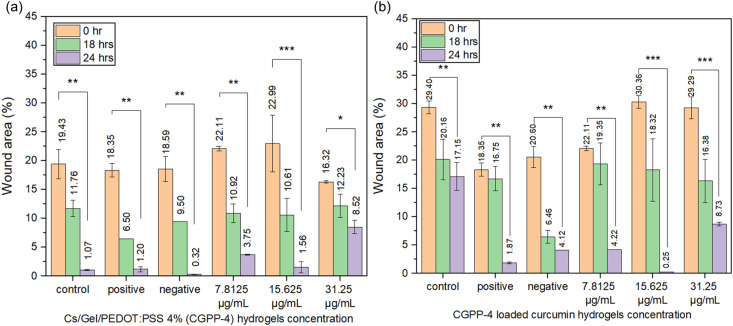
The wound area plots of keratinocyte tissue treated with (a) CGPP-4 hydrogels and (b) curcumin-loaded CGPP4 hydrogels at 0, 18 and 24 hours of treatment. (*P < 0.5, **P < 0.05, and ***P < 0.005).

Scratch-wound assays conducted over 0, 18, and 24 h demonstrated progressive wound closure in all CGPP-4-treated groups (Fig. 10a), as well as in curcumin-loaded CGPP-4 groups (Fig. 10b), indicating that the hydrogel system supports keratinocyte migration over time. These observations are best interpreted as correlative outcomes arising from multiple interacting material properties, rather than being driven by a single parameter. Specifically, the porous architecture of CGPP-4 likely facilitates cell migration and nutrient transport, while its hydrophilic chitosan–gelatin matrix promotes cell adhesion through ECM-like biochemical cues. The presence of PEDOT:PSS introduces mixed ionic–electronic conductivity, which may influence the local electrochemical environment and contribute to cell behavior in conjunction with these physical and chemical factors.^86^ However, the independent contribution of conductivity was not isolated in this study and is therefore discussed as a supportive, rather than dominant, factor.

To further explore the multifunctionality of the hydrogel, curcumin was incorporated into CGPP-4 as a model bioactive agent.^87,88^ Although detailed release kinetics were not investigated, the inclusion of curcumin enabled evaluation of the hydrogel as a combined scaffold and localized delivery platform. Among the tested groups, curcumin-loaded CGPP-4 at 15.625 µg mL^−1^ exhibited the most pronounced wound closure, achieving near-complete re-epithelialization (SI 7, S7). This enhanced response likely reflects a synergistic interaction between the hydrogel matrix and the well-established anti-inflammatory and antioxidant activities of curcumin, rather than a direct effect of conductivity alone.^89^ The improved wound-closure behavior can thus be attributed to the combined influence of multiple factors, including hydrogel porosity, surface wettability, swelling behavior, polymer composition, and localized curcumin availability. The chitosan–gelatin matrix provides a biomimetic ECM-like environment that supports keratinocyte attachment and migration,^4^ while agarose-templated porosity enhances mass transport and cell infiltration. The presence of PEDOT:PSS and curcumin may further modulate cellular responses through electrochemical and biochemical pathways, respectively.

**Fig. 11:**
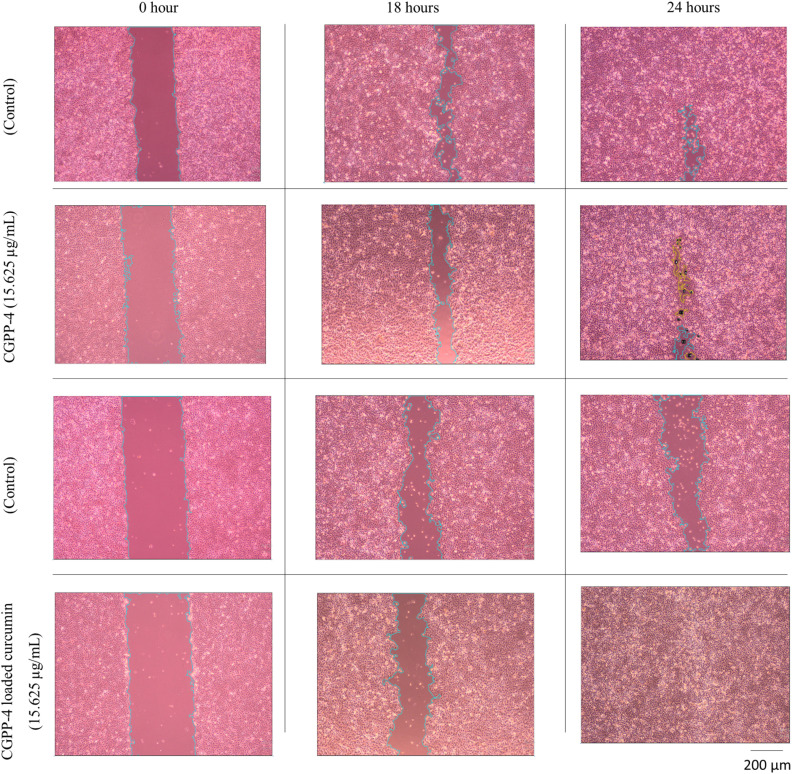
*In vitro* wound healing (scratch) assay of keratinocyte monolayers treated with the CGPP hydrogels. Representative optical micrographs show wound closure at 0, 18, and 24 h for control cells and cells treated with CGPP-4 (15.625 µg mL^−1^), with and without curcumin loading. The initial scratch area at 0 h is clearly visible in all groups. Over time, progressive cell migration toward the wound gap is observed, as indicated by the narrowing of the scratched region (outlined). Compared to the control, CGPP-4-treated cells exhibit accelerated wound closure, with a more pronounced reduction in wound width at 18 h and near-complete closure at 24 h. The curcumin-loaded CGPP-4 further enhances keratinocyte migration, resulting in almost complete wound closure by 24 h. Scale bar: 200 µm.

Interestingly, while both 15.625 and 31.25 µg mL^−1^ curcumin-loaded CGPP-4 promoted wound closure (Fig. 10b), the lower concentration produced superior outcomes (Fig. 11), suggesting the existence of an optimal therapeutic window. Excessive curcumin loading may introduce cytotoxic or osmotic stress, whereas insufficient levels may be inadequate to activate regenerative pathways. Overall, these findings demonstrate that CGPP-4 functions as a multifactorial wound-healing platform, in which electrical conductivity, material structure, and bioactive molecule delivery collectively contribute to enhanced keratinocyte migration and proliferation. The incorporation of PEDOT:PSS introduces mixed ionic–electronic conductivity, allowing the hydrogel to support charge transport *via* coupled electronic conduction along the PEDOT-rich domains and ionic conduction through the aqueous phase of the polymer network. Under physiological conditions, this dual conduction mechanism is likely modulated by ion redistribution and polymer chain rearrangement, as reflected by the time-dependent impedance changes observed during PBS incubation. While no new chemical structures are formed during cell incorporation, the presence of PEDOT:PSS may locally alter the electrochemical microenvironment at the cell–hydrogel interface, potentially influencing signal transduction pathways associated with keratinocyte migration. These effects are proposed to act synergistically with the hydrogel's physicochemical properties, contributing to the observed enhancement in wound closure without attributing cellular responses solely to electrical conductivity. While causal attribution to individual parameters remains beyond the scope of this study, the results support the potential of CGPP-based hydrogels as adaptable wound-dressing systems for bioelectronic and regenerative applications.

## 4. Conclusions

This study successfully developed chitosan/gelatin/PEDOT:PSS (CGPP) hydrogels with mixed ionic–electronic conduction and elucidated their coupled swelling, degradation and release mechanisms under physiological conditions. The morphological, crystallographic, and spectroscopic analyses confirm that PEDOT:PSS was successfully incorporated into the chitosan/gelatin hydrogel network and significantly influenced its structural organization. FESEM observations demonstrated that all hydrogels preserved a porous, interconnected, and cell-permissive architecture; however, increasing the PEDOT:PSS content resulted in progressively smaller and more compact pore domains. This reduction in pore size is attributed to both the inherent semi-crystalline packing of PEDOT:PSS and the formation of S–N sulfenamide linkages between the thiophene ring of PEDOT and the amino groups of chitosan, which introduce localized rigidity and densification within the polymer network. These complementary characterizations demonstrate that PEDOT:PSS incorporation not only modifies the hydrogel's nanoscale architecture and chain packing but also forms new chemical interactions that reinforce the structural integrity of the composite system. The resulting CGPP hydrogels exhibit a unified, miscible network in which chitosan, gelatin, and PEDOT:PSS are molecularly integrated. *In vitro* studies showed that increasing PEDOT:PSS content enhanced hydrophilicity and water affinity due to the hygroscopic sulfonate groups of PSS, leading to greater swelling and accelerated mass loss. The degradation behaviour strongly correlated with water uptake, with highly hydrated gels, particularly CGPP-6, exhibiting the fastest disintegration. Beyond hydrolytic softening, we propose that persistent weak ionic interactions between protonated chitosan (NH_3_^+^) and gelatin carboxylates (COO^−^) act as competing forces that partially override crosslinking density, promoting gradual network loosening over time.

ATR-FTIR and UV-vis findings confirm the partial retention of chitosan, gelatin, and PEDOT:PSS at higher loadings, alongside solubilization of conjugated and aromatic degradation products, indicating dynamic rearrangement and gradual erosion of the composite matrix. PEDOT:PSS incorporation markedly enhanced surface hydrophilicity as reflected by decreased contact angles and contributed to improved swelling and biointerface properties, which are essential for tissue–material interactions. Electrochemical impedance spectroscopy further revealed an optimal conductive window at moderate PEDOT:PSS levels (3–4 vol%), where stable percolation networks sustained physiological-range conductivity even after one week of hydrolytic stress, before declining by two weeks due to leaching and increased PEDOT grain spacing from water-rich PSS domains. Despite these degradative changes, the hydrogels retained semi-conductive behavior and favourable wettability, supporting their continued ability to deliver electrical cues while undergoing controlled biodegradation.

Overall, the findings demonstrate that the CGPP hydrogel system, particularly the CGPP-4 formulation, successfully integrates electrical conductivity, biocompatibility, and bio-functional performance to support tissue regeneration. The initial cytotoxicity screening confirmed that all CGPP variants maintained cell viability above 80%, validating their safety for biomedical use, while the incorporation of PEDOT:PSS contributed beneficial electroactive properties without inducing harmful cellular responses. CGPP-4, selected for its conductivity closely matching that of human skin and its stability under physiological conditions, promoted robust keratinocyte proliferation at biologically relevant concentrations and enabled effective wound closure in scratch assays. Furthermore, the addition of curcumin enhanced this regenerative response, with 15.625 µg mL^−1^ emerging as the optimal dose for achieving complete re-epithelialization through a synergistic interplay between electroconductive signaling, antioxidant protection, and ECM-mimetic structural support. Collectively, these results establish CGPP-4 as a multifunctional hydrogel platform capable of delivering both electrical and biochemical cues to accelerate wound healing, positioning it as a promising candidate for next-generation bioelectronic wound dressings.

While the CGPP hydrogel system demonstrates strong *in vitro* bioelectronic and regenerative performance, further studies are required to support clinical translation. *In vivo* evaluation is essential to validate biocompatibility, degradation behavior, and conductive stability under complex physiological conditions involving enzymatic activity, immune responses, and mechanical stress. Particular attention should be given to the long-term fate and biosafety of PEDOT:PSS degradation products. Future work should also extend beyond two-dimensional assays toward three-dimensional skin models and full-thickness wound models to better capture tissue complexity, vascularization, and inflammatory dynamics. Optimization of mechanical robustness, adhesion, and moisture retention will be critical for practical wound-dressing applications. From a translational perspective, scalability, sterilization compatibility, and batch-to-batch reproducibility must be addressed to ensure consistent performance. Finally, integration with external or wearable electrical stimulation systems represents a promising pathway toward advanced bioelectronic wound dressings capable of delivering controlled therapeutic cues. Collectively, these efforts will be pivotal in advancing CGPP-4 from a laboratory platform toward clinically relevant regenerative applications.

## Author contributions

Conceptualization, D. A. A. R., M. M. M., Z. M. S. and K. K. S.; methodology, D. A. A. R., M. M., N. S., Z. M. S. and H. O.; formal analysis, D. A. A. R., M. M. M., N. S. and M. M.; writing—original draft preparation, D. A. A. R.; writing—review and editing, D. A. A. R., M. M. M., K. K. S., N. S., R. R., H. O., N. H. S., and M. I. M. G.; visualization, D. A. A. R., M. M. M., K. K. S., and N. H. S.; and supervision, M. M. M., H. O., R. R., M. I. A. H., and Z. M. S. All authors have read and agreed to the published version of the manuscript.

## Conflicts of interest

There are no conflicts of interest to declare.

## Supplementary Material

RA-016-D5RA09790H-s001

## Data Availability

The data that support the findings of this study are available from the corresponding author upon reasonable request. Supplementary information is available. See DOI: https://doi.org/10.1039/d5ra09790h.
