# Supplementary material for: Synthesis and characterization of electroactive chitosan/gelatin/PEDOT:PSS hydrogels with mixed ionic–electronic conductivity for potential wound healing applications
Source: RSC Adv. 2026 Feb 9;16(9):7943–65. doi: 10.1039/d5ra09790h (PMC12884481; doi:10.1039/d5ra09790h)
Supplement: RA-016-D5RA09790H-s001 [file RA-016-D5RA09790H-s001.pdf]

Supplementary Information (SI) for RSC Advances.
This journal is © The Royal Society of Chemistry 2026

Supplementary 1

Illustration of linear scratch on 6-well plate for wound healing assay.

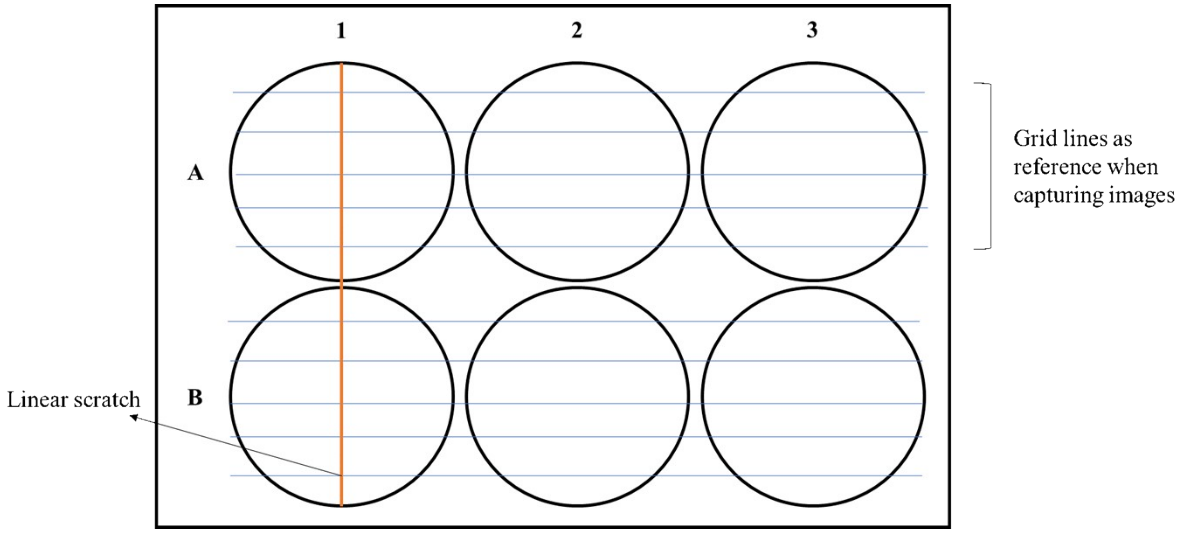

Supplementary 2

The Measured Biodegradation Rate of (CGPP) Hydrogels.

CGPP hydrogels type	Biodegradation rate (g min^{-1})
CGPP-0	0.0008
CGPP-1	0.0012
CGPP-2	0.0014
CGPP-3	0.0015
CGPP-4	0.0014
CGPP-5	0.0020
CGPP-6	0.0026

Supplementary 3

The Calculated Concentration of Hydrogels Released in PBS Solutions After 14 Days of Incubation

CGPP hydrogels type released in PBS	Absorbance (a.u.)	Calculated concentration (mol/cm^3)
CGPP-0	0.3348	0.0018
CGPP-1	0.3767	0.0395
CGPP-2	0.4172	0.0760
CGPP-3	0.3801	0.0425
CGPP-4	0.4125	0.0718
CGPP-5	0.3524	0.0177

CGPP-6	0.3771	0.0399

Supplementary 4

The measured mixed ionic–electronic conductivity values of CGPP hydrogels (mean ± standard deviation) before incubation and after 1 and 2 weeks of incubation under physiological conditions.

**Hydrogels**	**Mean**	**Standard deviation**
	**Before Incubation**	
CGPP-0	1.19×10^{-3}	$\pm 0.07 \times 10^{-3}$
CGPP-1	8.32×10^{-4}	$\pm 0.05 \times 10^{-4}$
CGPP-2	4.51×10^{-4}	$\pm 0.03 \times 10^{-4}$
CGPP-3	1.51×10^{-3}	$\pm 0.09 \times 10^{-3}$
CGPP-4	2.46×10^{-3}	$\pm 0.15 \times 10^{-3}$
CGPP-5	5.49×10^{-4}	$\pm 0.04 \times 10^{-4}$
CGPP-6	3.78×10^{-4}	$\pm 0.03 \times 10^{-4}$
	**After 1-week incubation**	
CGPP-0	1.13×10^{-3}	$\pm 0.08 \times 10^{-3}$
CGPP-1	6.75×10^{-4}	$\pm 0.05 \times 10^{-4}$
CGPP-2	4.21×10^{-4}	$\pm 0.03 \times 10^{-4}$
CGPP-3	1.49×10^{-3}	$\pm 0.10 \times 10^{-3}$
CGPP-4	2.49×10^{-3}	$\pm 0.18 \times 10^{-3}$
CGPP-5	5.41×10^{-4}	$\pm 0.04 \times 10^{-4}$
CGPP-6	3.20×10^{-4}	$\pm 0.03 \times 10^{-4}$
	**After 2 weeks incubation**	
CGPP-0	8.47×10^{-4}	$\pm 0.09 \times 10^{-4}$
CGPP-1	8.65×10^{-4}	$\pm 0.07 \times 10^{-4}$
CGPP-2	4.21×10^{-4}	$\pm 0.04 \times 10^{-4}$
CGPP-3	3.45×10^{-4}	$\pm 0.05 \times 10^{-4}$
CGPP-4	4.74×10^{-4}	$\pm 0.06 \times 10^{-4}$
CGPP-5	4.09×10^{-4}	$\pm 0.04 \times 10^{-4}$
CGPP-6	2.69×10^{-4}	$\pm 0.03 \times 10^{-4}$

Supplementary 5

The images of cells captured from microscope representing the condition of cells after 24 hours of CGPP hydrogels treatment

control 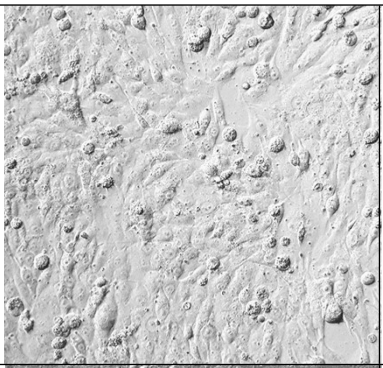	CGPP-0 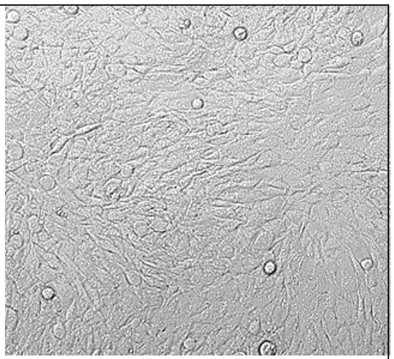
CGPP-1 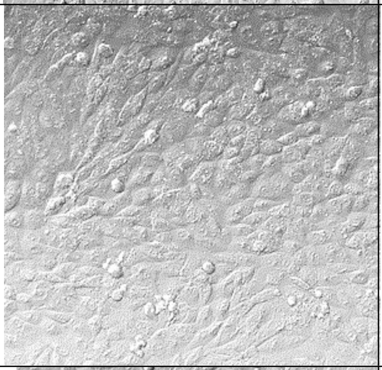	CGPP-2 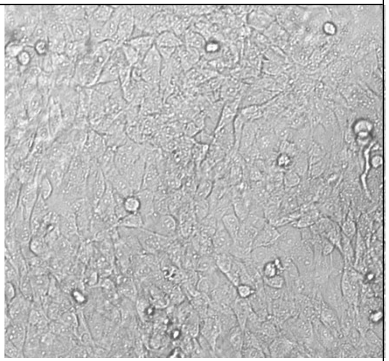
CGPP-3 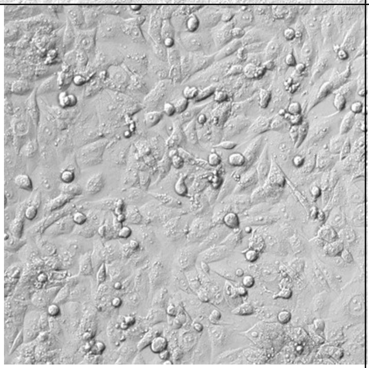	CGPP-4 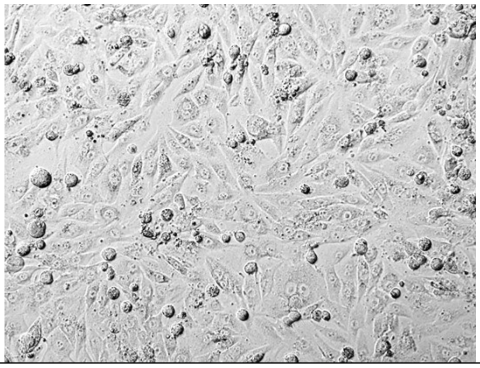
CGPP-5 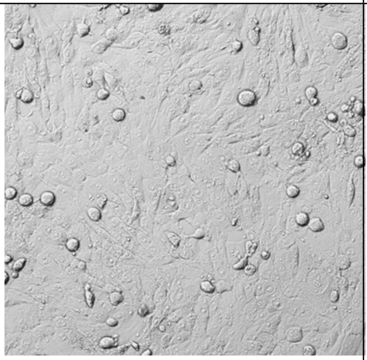	CGPP-6 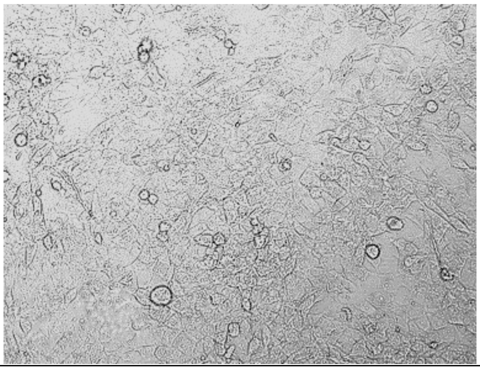

Supplementary 6

The cellular morphology of cells captured from optical microscope after a 72-Hours incubation treatment with CGPP-4 hydrogels.

blank 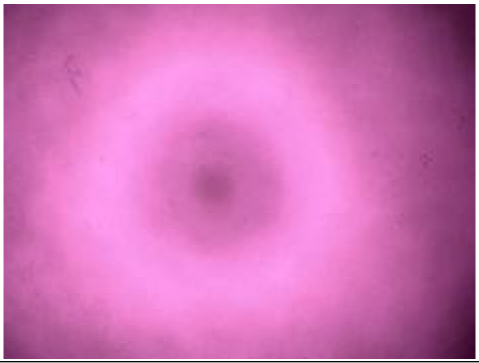	positive control 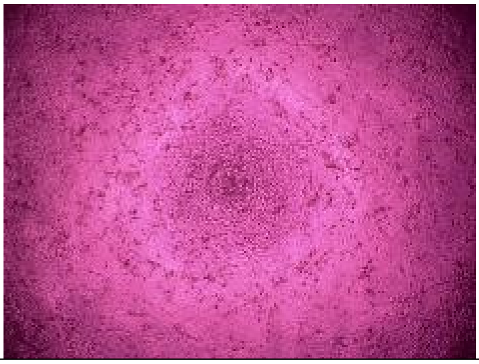
7.8125 mg/mL 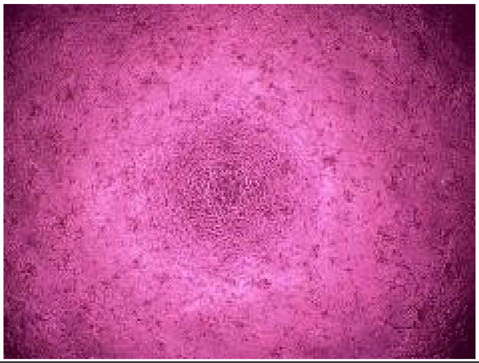	15.625 mg/mL 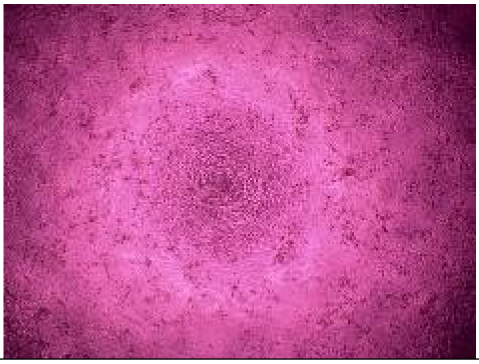
31.25 mg/mL 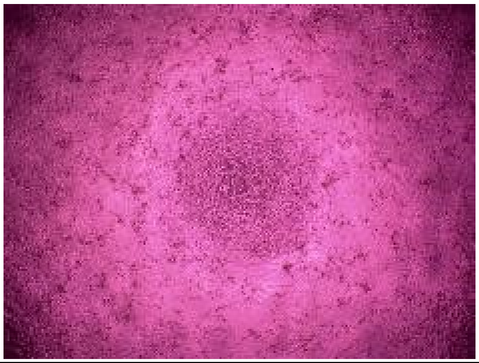	62.5 mg/mL 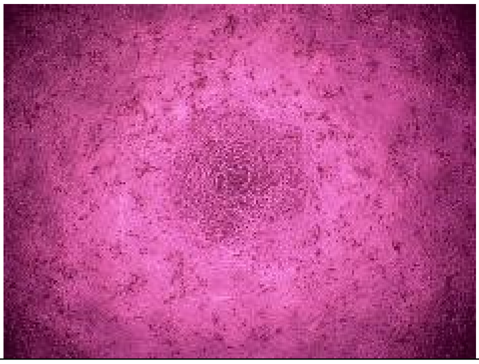
125.0 mg/mL 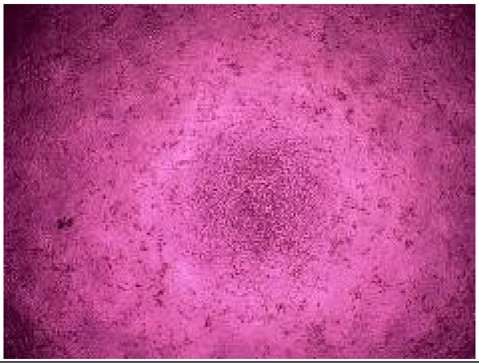	250.0 mg/mL 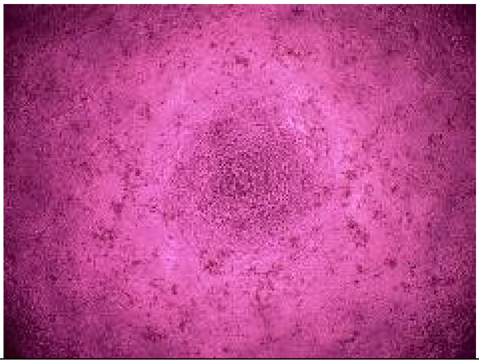

500.0 mg/mL	1000.0 mg/mL
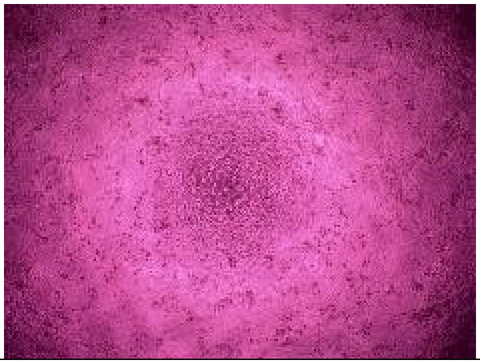	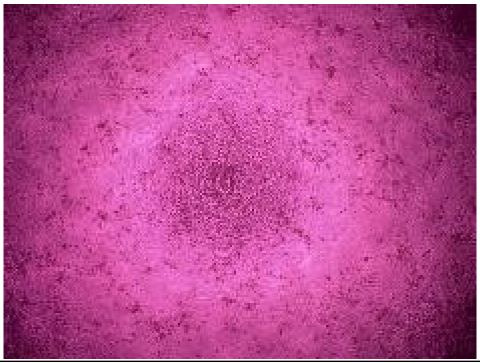

Supplementary 7

The cellular morphology of keratinocytes tissues underwent healing process captured from optical microscope after at 0, 18 and 24-Hour incubation treatment with CGPP-4 hydrogels.

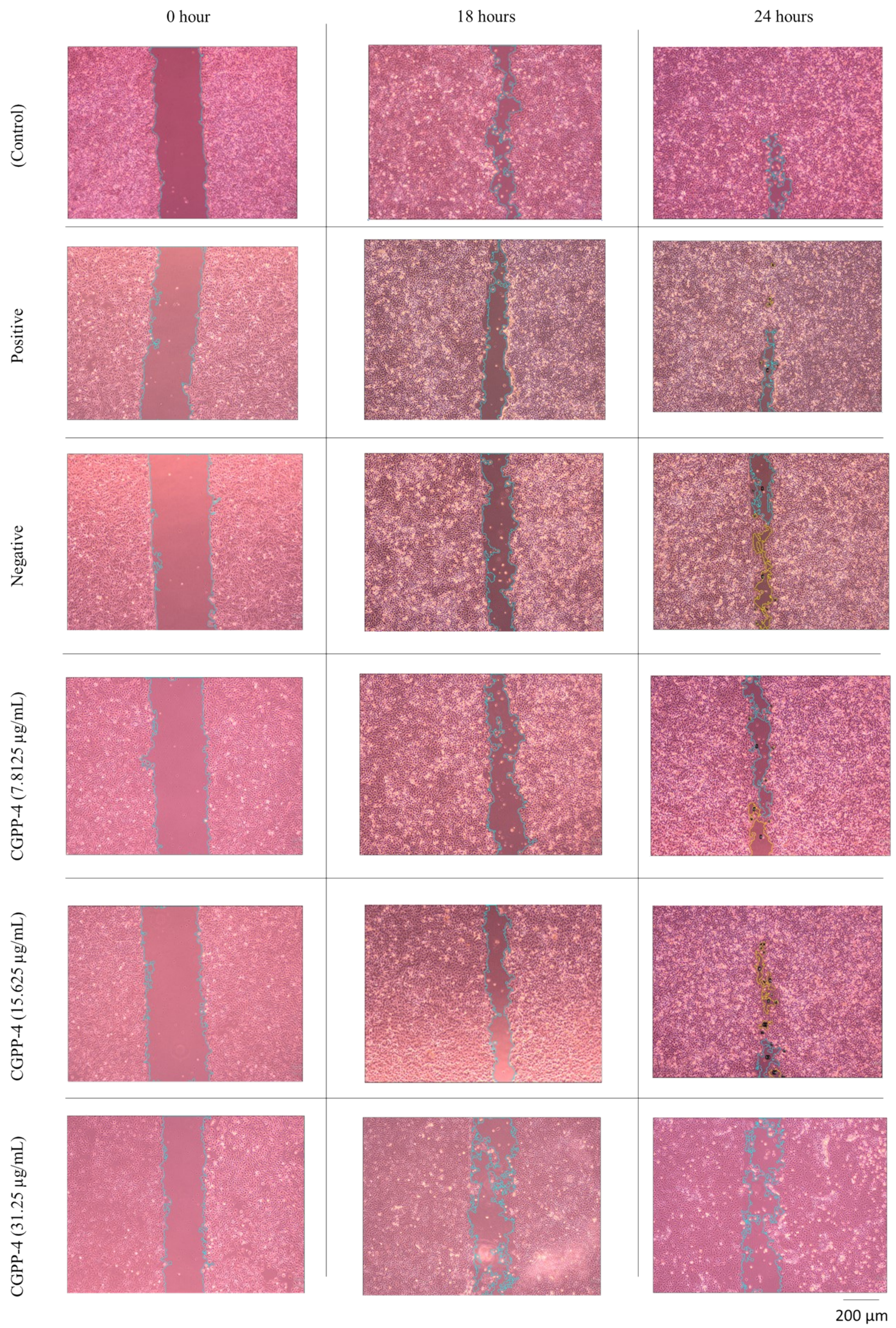

0 hour
18 hours
24 hours
(Control)
Positive
Negative
CGPP-4 (7.8125 µg/mL)
CGPP-4 (15.625 µg/mL)
CGPP-4 (31.25 µg/mL)
200 µm

The cellular morphology of keratinocytes tissues underwent healing process captured from optical microscope after at 0, 18 and 24-Hour incubation treatment with CGPP-4-loaded curcumin hydrogels.

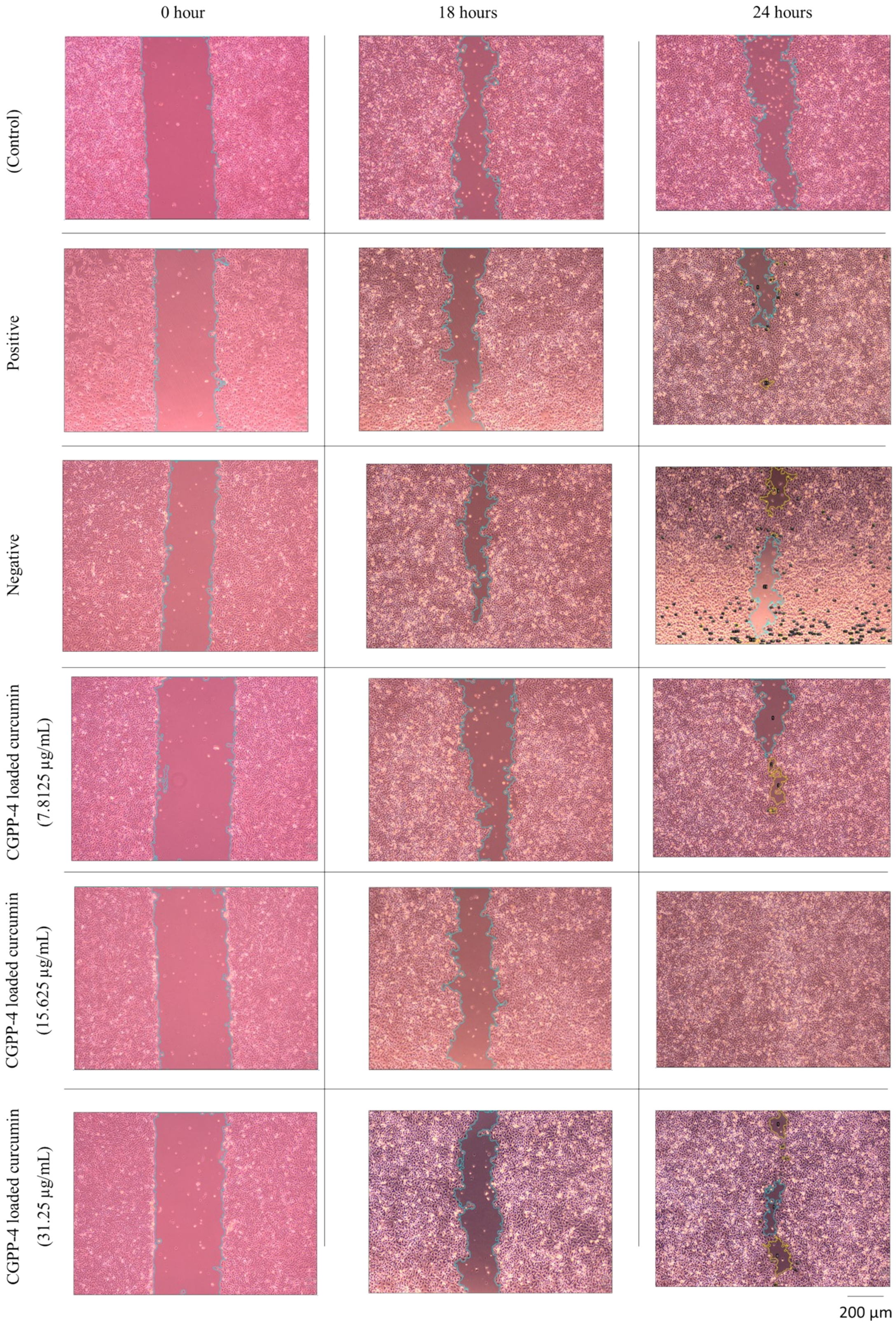
0 hour
18 hours
24 hours
(Control)
Positive
Negative
CGPP-4 loaded curcumin (7.8125 µg/mL)
CGPP-4 loaded curcumin (15.625 µg/mL)
CGPP-4 loaded curcumin (31.25 µg/mL)
200 µm
